# Cortex-wide neural dynamics predict behavioral states and provide a neural basis for resting-state dynamic functional connectivity

**DOI:** 10.1016/j.celrep.2023.112527

**Published:** 2023-05-26

**Authors:** Somayeh Shahsavarani, David N. Thibodeaux, Weihao Xu, Sharon H. Kim, Fatema Lodgher, Chinwendu Nwokeabia, Morgan Cambareri, Alexis J. Yagielski, Hanzhi T. Zhao, Daniel A. Handwerker, Javier Gonzalez-Castillo, Peter A. Bandettini, Elizabeth M.C. Hillman

**Affiliations:** 1 Mortimer B. Zuckerman Mind Brain Behavior Institute and Department of Biomedical Engineering, Columbia University, New York, NY, USA; 2 Section on Functional Imaging Methods, Laboratory of Brain and Cognition, National Institute of Mental Health, National Institutes of Health, Bethesda, MD, USA; 3 Functional MRI Core Facility, National Institute of Mental Health, National Institutes of Health, Bethesda, MD, USA; 4 Department of Radiology, Columbia University Irving Medical Center, New York, NY, USA; 5 These authors contributed equally; 6 Lead contact

## Abstract

Although resting-state functional magnetic resonance imaging (fMRI) studies have observed dynamically changing brain-wide networks of correlated activity, fMRI’s dependence on hemodynamic signals makes results challenging to interpret. Meanwhile, emerging techniques for real-time recording of large populations of neurons have revealed compelling fluctuations in neuronal activity across the brain that are obscured by traditional trial averaging. To reconcile these observations, we use wide-field optical mapping to simultaneously record pan-cortical neuronal and hemodynamic activity in awake, spontaneously behaving mice. Some components of observed neuronal activity clearly represent sensory and motor function. However, particularly during quiet rest, strongly fluctuating patterns of activity across diverse brain regions contribute greatly to interregional correlations. Dynamic changes in these correlations coincide with changes in arousal state. Simultaneously acquired hemodynamics depict similar brain-state-dependent correlation shifts. These results support a neural basis for dynamic resting-state fMRI, while highlighting the importance of brain-wide neuronal fluctuations in the study of brain state.

## In brief

Shahsavarani et al. investigate the dynamic correlation structure of cortex-wide neuronal activity and hemodynamics in awake mice using wide-field optical mapping. They demonstrate a relationship between neuronal correlation dynamics, arousal level, and behavior that is reproduced by hemodynamic correlations. Results support a neural basis for fMRI-based dynamic functional connectivity mapping.

## Graphical Abstract

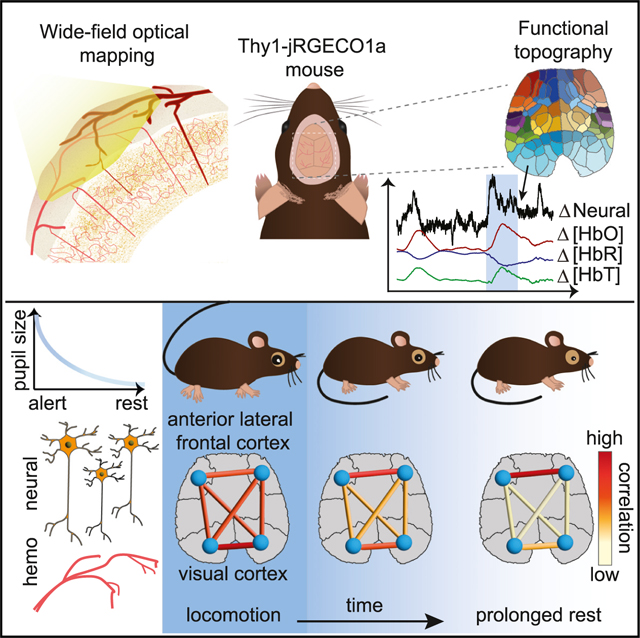

## INTRODUCTION

Functional brain imaging studies have traditionally focused on changes in brain activity evoked by specific stimuli or tasks. However, analysis of ongoing “resting-state” activity, recorded using functional magnetic resonance imaging (fMRI) has revealed interregional correlation patterns among spatially distinct brain regions, depicting the brain as a collection of functionally interconnected networks.^1^ Furthermore, these correlation patterns have been found to change over time,^2–7^ and the field of resting-state dynamic functional connectivity (rs-dFC) has emerged to investigate the dynamics of these networks and characterize their relationship to brain states.^8–11^

Analyses of dynamic changes in functional connectivity during an fMRI session have revealed potential biomarkers of neurological and psychiatric disease.^12,13^ However, results have not yet been sufficiently robust and reliable to achieve clinical significance. One factor hindering the interpretation of results, and refinement of analysis methods, is the dependence of fMRI signals on brain hemodynamics rather than neuronal activity. Meanwhile, methods capable of capturing and decoding properties of real-time brain-wide resting-state neuronal activity have been limited. Insights into the nature and properties of the brain-wide neural activity underlying signals detected and analyzed in fMRI rs-dFC are needed, both to reconcile our understanding of this activity across scales and species and to be able to leverage observations of this activity to understand the human brain in health and disease.

Wide-field optical mapping (WFOM) is a technique that can image both neuronal activity and hemodynamics across the entire dorsal cortex of the awake, behaving mouse.^14^ Leveraging fluorescent calcium indicators expressed in excitatory neurons, WFOM has previously been used to demonstrate that resting-state hemodynamics in the brain of awake mice can be largely predicted by baseline fluctuations in the excitatory neuronal activity.^15^ Here, we leverage improved, red-shifted calcium indicators in cortical neurons to visualize these neuronal patterns with a higher spatiotemporal resolution. We combine this imaging of neuronal activity with simultaneous mapping of cortical hemodynamics and detailed recordings of spontaneous behavior and pupil dynamics in awake mice to analyze the relationship between real-time brain activity, brain state, and behavior.

We observed robust, real-time patterns of ongoing neuronal activity across the brain that exhibited rich spatiotemporal correlation structures, while also including clear functional representations of sensory and motor behaviors. Correlations between neural activity in different cortical areas exhibited relatively stable patterns across animals when averaged across 10 minute epochs and reveal a compelling functional cortical topography. However, a moving-window correlation approach confirmed that neural correlation patterns shift and change over time, consistent with fMRI rs-dFC. Analysis of the relationship between different correlation states and behavior revealed consistent changes in correlation, not only before, during, and after spontaneous locomotion but also with varying arousal levels during quiescence or rest. Reduced arousal level was accompanied by an increase in the amplitude of neural fluctuations, predominantly in the anterior lateral frontal cortex, reducing the correlation between anterior and posterior brain regions. These resting-state neuronal fluctuations cannot be detected in trial-averaged data owing to their random phase and do not correspond to any discernable sensory input or motor output.

Repeating the same analysis on simultaneously recorded cortical hemodynamics data revealed that similar behavioral state transitions could be detected. Comparison of neural and hemodynamic signals confirmed that hemodynamics provide a temporally low-pass filtered representation of neuronal activity. Analysis of the frequency-dependence of correlations found relatively good agreement between neuronal and hemodynamic correlation patterns at lower frequencies. Importantly, neuronal correlation patterns remained consistent even when neuronal signals were high-pass filtered to retain frequencies exceeding those represented by hemodynamics. This finding supports the broadband nature of the neuronal activity underlying dynamic correlations and explains why lower-frequency hemodynamics are able to capture similar correlation features and transitions.

Our results support converging evidence that dynamic, brain-wide patterns of neural activity are fundamental to understanding brain states and help to resolve our understanding of the structure and temporal variability of functional connectivity across species and scales.

## RESULTS

Neural and hemodynamic activity across the dorsal cortex was simultaneously recorded at 20 frames per second using WFOM in five thinned-skull, awake, spontaneously behaving head-fixed Thy1-jRGECO1a transgenic mice expressing a red-shifted calcium indicator in neurons^16,17^ (Figure S1). Hemodynamic activity was imaged using green and red diffuse reflectance, which was mathematically converted into changes in oxyhemoglobin (Δ[HbO]), deoxyhemoglobin (Δ[HbR]), and total hemoglobin (ΔHbT) concentration (Δ[HbT]=Δ[HbO]+Δ[HbR]).^14^ Reflectance data were also used to generate a correction for the jRGECO1a fluorescence data to account for the time-varying absorption effects of hemoglobin (see STAR Methods).

**Figure 1.**
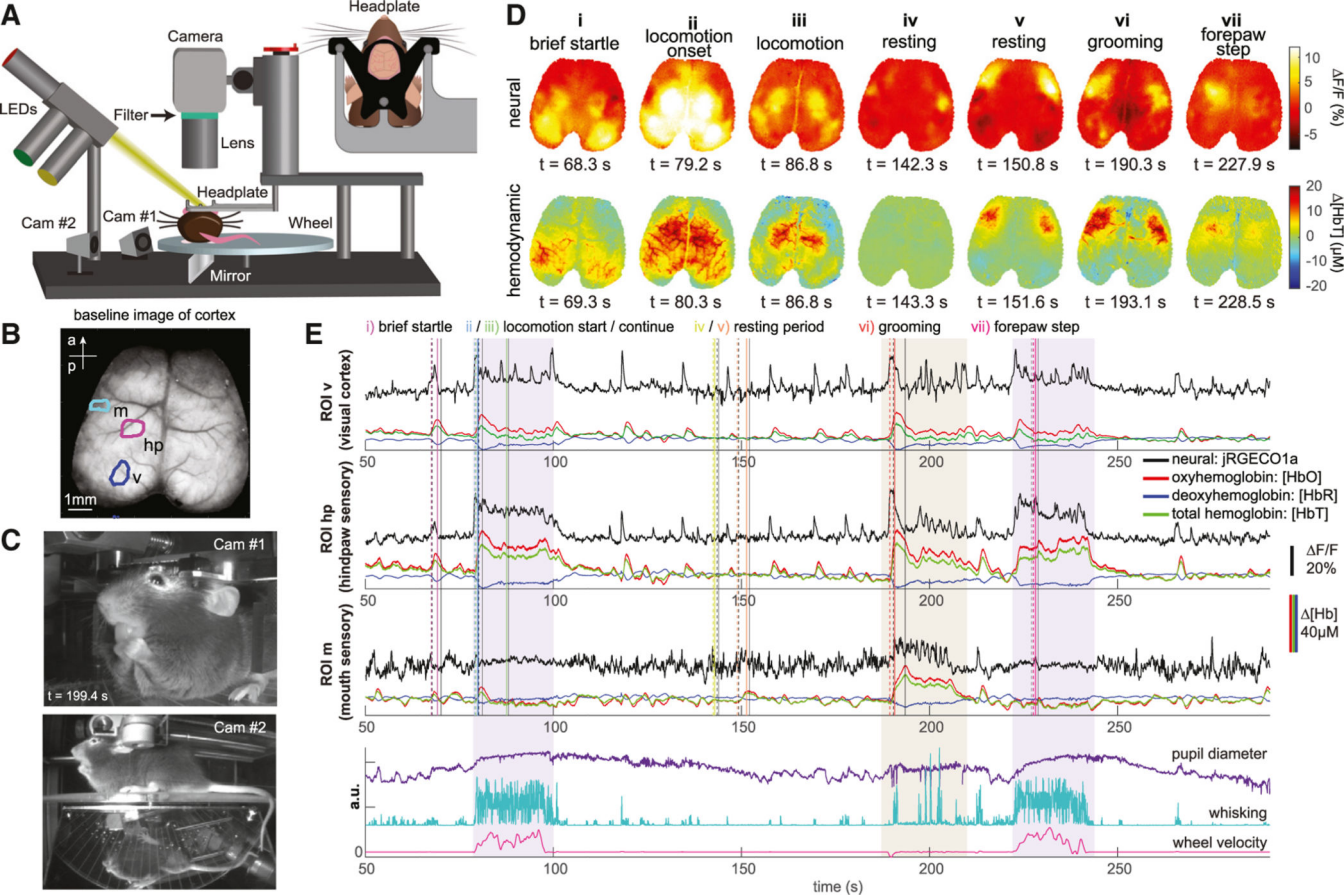
Imaging setup and raw data images and signals (A) Schematic of wide-field optical mapping (WFOM) system. The mouse is positioned on a freely moving horizontal wheel with dual-camera behavioral monitoring. Inset shows a head-fixation plate surrounding the thinned-skull cranial window. (B) WFOM camera field of view showing raw jRGECO1a image. (a, anterior; p, posterior). (C) Behavior camera 1 captures the face, mouth, pupil, and forepaws. Camera 2 captures the side and underside of the mouse via an angled mirror. (D) Top, neural (Δ% fluorescence change after hemodynamic correction) and bottom, total hemoglobin (ΔHbT) images corresponding to specific events listed (frame times for each event are indicated by color-coded [neural] and black [hemodynamic] solid vertical lines in [E], with subtracted prior reference frames shown as dashed lines). See Figure S2 for cortical atlas. (E) Neural and hemodynamic time courses extracted from mouth (m), forepaw (fp), and visual (v) ROIs indicated in (B). Bottom plot shows simultaneously recorded pupil diameter, whisking and locomotion, including a period of grooming. Hindpaw regions show strong activity during locomotion, while hindpaw and mouth regions respond during grooming. Visual cortex shows strong but less sustained activity for both events. Small startle responses can also be seen. The coupling between neural activity and hemodynamics, increasing HbT and HbO and decreasing HbR, can be clearly observed for small and large events. See also Figure S1 and Videos S1 and S2.

The mice were free to spontaneously move, walk, and run on a lightweight horizontal rotating, transparent acrylic wheel and were each imaged longitudinally for at most 10 repeated 10-min recording sessions within up to eight experimental sessions spanning 8 weeks. To measure changes in behavior and physiological arousal, variables such as whisking, pupil diameter, and locomotion were extracted from simultaneous video monitoring of the mouse and wheel movements as illustrated in Figures 1A–1C.

### Cortical representations of sensory and motor function

Figures 1D and 1E shows examples of raw data including cortical images and time courses extracted from different regions of interest (ROIs) in the cortex. Figure S2 verifies identified cortical regions and compares them with a standard atlas, while Videos S1 and S2 show examples of real-time neural and hemodynamic data.

During spontaneous locomotion, sensory forepaw and hindpaw regions can be seen to exhibit strong increases in neuronal activity (Figure 1D ii, iii, and vii) and an associated hemodynamic response (increasing [HbT] and [HbO], and decreasing [HbR]) (Figure 1E). A similar pattern is seen for transient activation of the visual cortex at locomotion onset (Figure 1D ii), while smaller startle or twitching events show transient activations of broader sensory regions (Figure 1D i). Grooming shows selective activation of the mouth and forepaw sensory areas (Figure 1D vi).

In addition to neuronal events related to sensory and motor function, distinct fluctuations in neuronal activity and hemodynamics are also clearly visible during periods of rest when only minimal movements and sensory stimuli occur, particularly in more anterior regions (Figure 1D iv-v; Video S1). These activity patterns tend to be bilaterally symmetric for both neuronal activity and hemodynamics.

### Spatiotemporal clustering of neural data during rest reveals a consistent functional topography

**Figure 2.**
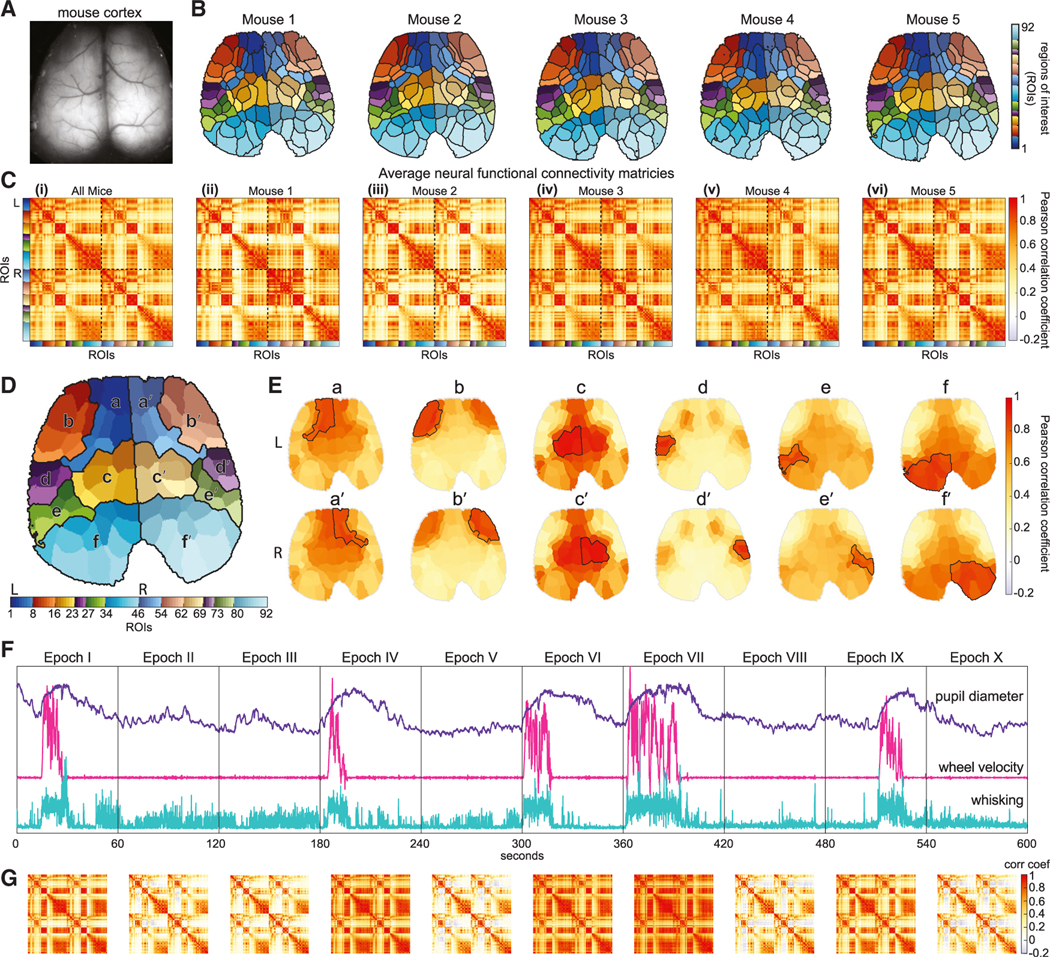
Functional topography of mouse cortex and long-epoch and dynamic neural correlation maps (A) Mouse cortex field of view. (B) Functional topographic parcellations of the cortex into 92 bilaterally symmetric ROIs using spatiotemporal clustering of resting-state neural activity for each mouse. (C) Long-epoch (10-min) correlation maps of neural activity from ROIs in (B) averaged across all recording sessions (i) over all mice (see Video S3) and (ii-vi) for each mouse. (D) To simplify visualization, ROIs presented in (B) were grouped into six bilateral larger clusters based on the similarity of their average correlations (a and b, putatively frontal/motor; c-e, somatosensory; f, visual). (E) Simplified cortical representations of the average long-epoch correlation map, with a-f and a′-f′ showing the mean of correlation values between each subgroup in (D) and all ROIs. (F and G) (F) Behavioral signals for a 10-min acquisition session with (G) showing corresponding time-varying correlation maps for each 1-min epoch I-X. See also Figure S3 and Videos S2, S3, and S4.

To explore spatiotemporal correlation patterns across the cortex, it is essential to have a robust frame of reference that enables comparisons over time and between mice. By applying spatiotemporal clustering methods to neural data, regions that are temporally correlated can be delineated. This analysis reveals striking, symmetric and reproducible cortical “functional topographies,” with finest detail observed when clustering is applied to data collected during periods of quiet rest. By applying a data-driven process to refine cluster centroids (see STAR Methods), it was possible to generate consistent cortical topographies with 92 ROIs across five mice (Figure 2B). Figure S3 demonstrates that this number of ROIs preserves a majority of the variance in the raw neural data.

This topographic parcellation is important for two main reasons. First, it provides a robust basis for downstream analysis of dimensionally reduced and registered WFOM data, and thus comparisons between mice. Second, these spatiotemporally derived functional parcellations reveal a consistent and anatomically recognizable topography of the cortex depicting well-defined, bilaterally symmetric regions based only on the functional dynamics of each region during rest. Although prior studies using mice expressing the green fluorescent calcium-sensitive indicator GCaMP have revealed consistent yet relatively coarse parcellations of the mouse cortex,^18,19^ the faster dynamics and higher spatial fidelity in red-shifted Thy1-jRGECO1a mice provided a significantly more detailed structure.^17^ Compared with a standard mouse brain atlas in Figure S2, this cortical topography clearly delineates sensory regions such as whisker, forepaw, mouth, hindpaw, and visual regions (see Video S2). However, it also reveals finer segmentation of regions across the frontal cortex that are generally not resolved in anatomically derived atlases of the mouse cortex.^20,21^

### Long-epoch interregional neural correlation patterns are consistent across mice

With this ROI framework in place, we could explore and compare correlation relationships between different cortical regions. Time courses were extracted from each ROI, and then correlation matrices were computed by calculating Pearson correlation coefficients (zero-lag correlation) between the time series of each ROI pair, resulting in a symmetric 92 × 92 correlation map. Figure 2C shows correlation maps calculated over full 10-min epochs, averaged over all recording sessions for each mouse. Videos S3 and S4 shows the average correlation map for all mice projected onto the cortex for each ROI in turn. To enable simplified visualization of this correlation map on the cortex, we clustered the average correlation map into 12 groups (six on each side of the brain) and display the average correlation within each of these sub-regions and the rest of the cortex in Figures 2D and 2E (see STAR Methods).

Although this average, long-epoch neural correlation pattern is relatively consistent across five mice (Figure 2C i-iv), if we split a 10-min recording into temporal segments of 1 min each with no overlaps, we observe that interregional correlation patterns vary across time (see Figure 2G). When comparing these changing correlation patterns with mouse behavior, we observe congruous patterns for epochs during which the animal is at rest (epochs II, III, V, VIII, and X) or actively engaged in locomotion (epochs I, IV, VI, VII, and IX).

### Variations in neural correlations over time predict behavioral states

**Figure 3.**
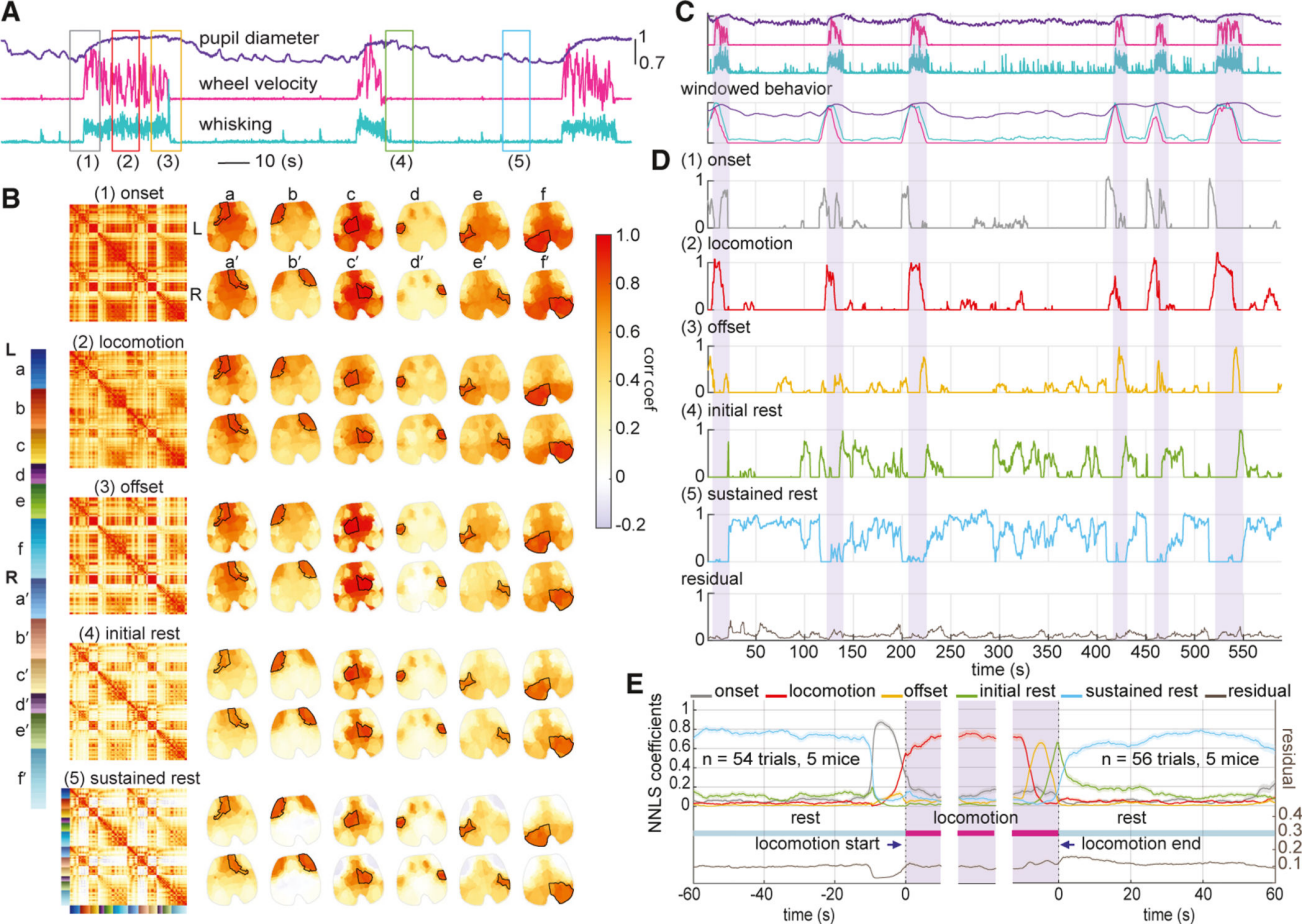
Neural correlation-based linear model of behavioral state (A) Plots of real-time behavioral time courses showing 3 locomotion bouts. Colored boxes (1–5) indicate epochs selected to represent (1) locomotion onset, (2) locomotion, (3) locomotion offset, (4) initial rest, and (5) sustained rest. (B) Average 10-s window neural correlation maps and cortical representations for each behavioral state defined in (A) for one example mouse. Brain maps represent the average correlation between each ROI and the outlined areas labeled as a-f and a′-f′. See also Video S3. (C) Mouse behavior for one example recording session, also plotted below smoothed with a 10-s window. (D) Corresponding non-negative least-squares (NNLS) coefficients for a fit to each 10-s moving-window correlation map using the behavioral state correlation maps in (B) as a basis set. Bottom plot shows fit residual (see STAR Methods). Shaded bars indicate locomotion bouts. (E) Average NNLS coefficients aligned over multiple locomotion events for five mice (n = 54 [onset], 47 [locomotion], and 56 [offset] epochs). Solid lines show average, while shaded bounds show standard errors (mean ± SEM). See also Figures S4 and S5 and Video S3.

It is not surprising that locomotion has a measurable effect on cortical correlations. Therefore, in orderto further probe correlation variability overtime, we next calculated inter-regional correlations within 10-s moving windows, focusing on five distinct types of epochs: (1) during the onset of locomotion, (2) during periods of sustained locomotion, (3) during locomotion offset, (4) during rest immediately after locomotion (initial rest), and (5) during rest from 40 to 50 s after locomotion (sustained rest) (see STAR Methods). Figure 3A depicts these epochs.

For each of the five mice, the average neural correlation map within each of these behavioral epochs was calculated using locomotion bouts selected from multiple days of recordings (see STAR Methods for details). The results for one example mouse, shown in Figure 3B, demonstrate that correlation maps across these behavioral states had distinctly different correlation patterns, both bilaterally and anterior-posteriorly. The results for the other mice, included in Figure S4, depict similar correlation patterns for each behavioral state across mice.

To determine how well these 5 average “behavioral correlation state maps” represent real-time neural correlation patterns in freely behaving mice, we used them as the basis set for a non-negative least-squares (NNLS) fit. Fit coefficients for each real-time 10-s-windowed correlation map represent the contribution of each behavioral correlation state map to the cortical correlation pattern at that moment in time (see STAR Methods).

Figures 3C and 3D show NNLS fit coefficients for one example recording session of neural activity. Fit coefficients for each behavioral correlation state align well with mouse behavior (i.e., predicting the onset, steady state, and offset of locomotion as well as initial rest and subsequent rest, all with low residuals). The relative lack of overlap between coefficients estimated by the model indicates robust switching between correlation states rather than over-fitting multiple states at one time. To quantify this concordance, we calculated the average behavioral correlation state coefficients with respect to the onset and offset of each locomotion bout (Figure 3E; see STAR Methods for more detail). These results confirm the consistency of correlation patterns within particular behavioral epochs. Video S4 shows real-time sequences of 10-s window correlation maps alongside cortical imaging data and mouse behavior.

### Differences between resting-state correlation patterns correlate with arousal level

**Figure 4.**
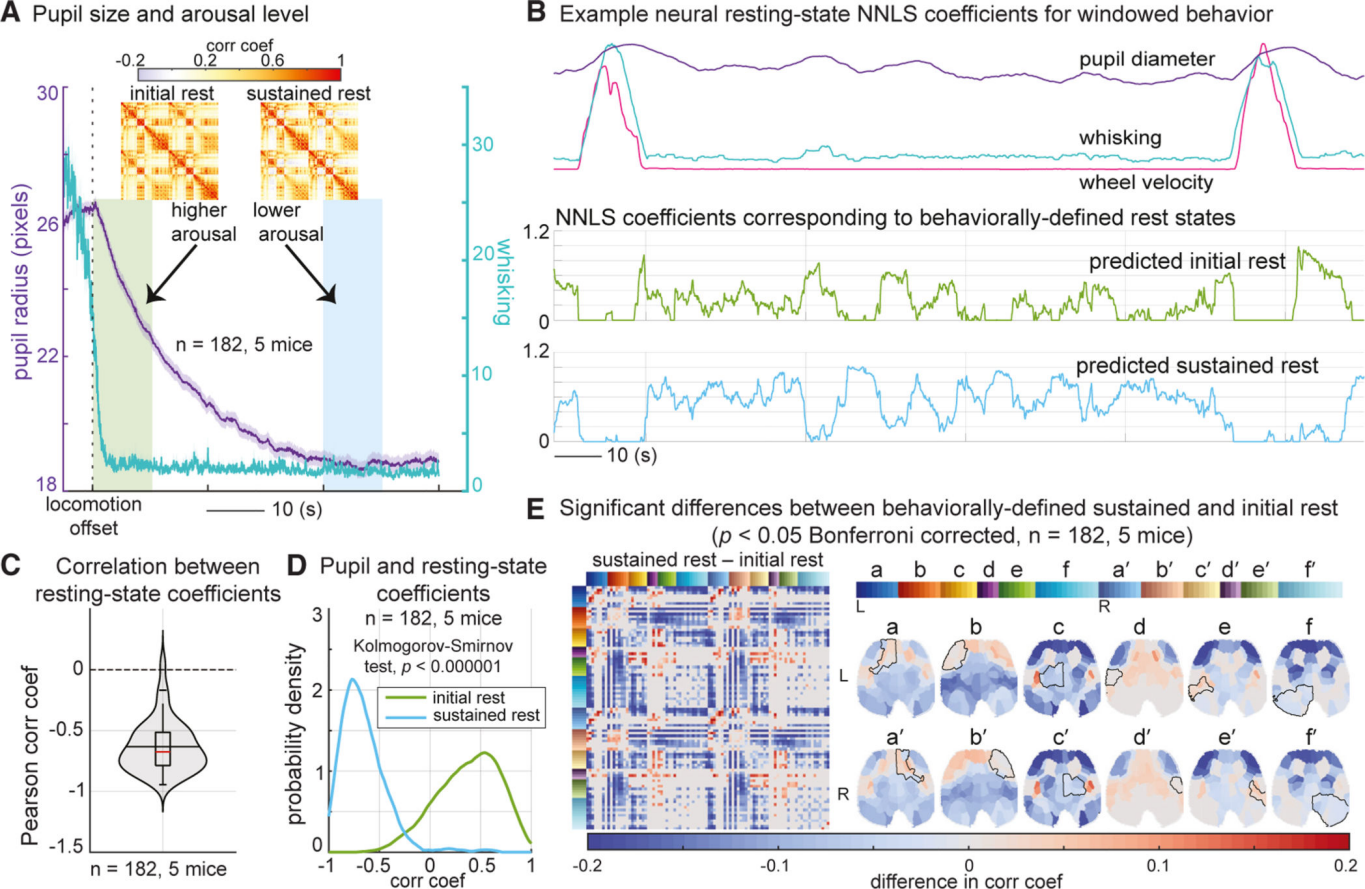
Differences in neural correlation states between initial and prolonged resting periods (A) Average whisking and pupil diameter aligned with running offset confirm higher and lower arousal states for initial (green bar) and prolonged (blue bar) rest epochs (n = 182, 5 mice), shown as mean ± SEM. Insets show correlation maps for initial-rest and sustained-rest states averaged across one example mouse. (B) Ten-second window smoothed behavior and NNLS coefficients for initial and sustained rest for one example resting-state epoch (with a locomotion bout before and after). Coefficients show first a transition from initial to sustained rest and then a reciprocal relationship as rest continues and whisker and pupil parameters vary spontaneously. (C) The reciprocal relationship between the initial-rest and sustained-rest NNLS coefficients is confirmed by negative correlation values during 182 resting (≥60 s long) epochs over five mice. (D) Probability distribution of correlation values between pupil diameter and NNLS coefficients for initial and sustained rest using kernel density estimation, calculated across the same 182 resting epochs. Distributions are significantly different (Kolmogorov-Smirnov test, p < 0.000001). (E) Correlation map differences between sustained- and initial-rest states (only significant differences are shown; Wilcoxon rank-sum test, p < 0.05, Bonferroni corrected, n = 182, five mice). Note the prominent decreased correlation between the anterior lateral frontal cortex and posterior brain regions.

Although changes in correlation structures between locomotion and rest are to be expected, we also detected more subtle differences between initial and prolonged rest. Consistent with previous reports,^22^ the average pupil diameter of our mice after locomotion offset followed a decreasing trend (Figure 4A), interpretable as gradually decreasing alertness or arousal level.^22,23^ Our “initial” and “prolonged” rest epochs were thus selected as the 10 s immediately after cessation of locomotion and 40 s later to capture higher and lower states of arousal.

In our NNLS modeling results, initial rest always precedes prolonged rest after locomotion offset (Figure 3D). However, after this initial transition, mice that continue to rest appear to transition in and out of the higher and lower arousal states. Figure 4B illustrates reciprocity between these two rest states, quantified by a strongly negative Pearson correlation (mean = −0.7, SD = 0.2) between the two coefficients across all rest periods of at least 60 s long across all mice (see Figure 4C). To explore the behavioral correlates of this resting-state switching, we calculated Pearson correlations between the resting-state coefficients and pupil diameter over the same rest periods across all mice. Figure 4D demonstrates the probability distribution of these correlation coefficients (fitting a kernel distribution). As expected, the initial-rest state is associated with larger pupil size and, therefore, a higher arousal level. In contrast, sustained rest is associated with smaller pupil size and hence lower arousal level (comparing the initial-rest and sustained-rest distributions, Kolmogorov-Smirnov test, k = 0.3, p < 1e–6). These results suggest that the neural correlation patterns are predictive of actual changes in the arousal level of the mouse.

Figure 4E examines the regions exhibiting significant differences in correlation between the sustained and initial rest across all mice using a Wilcoxon rank-sum test (p < 0.05, Bonferroni corrected). Mapped onto the cortex, we see that the most significant component is the anterior lateral frontal cortex, which becomes more desynchronized from posterior brain regions during sustained rest. Minimal differences in sensory and visual regions confirm that correlation changes do not simply reflect functional changes due to pupil dynamics or small whisking events.

To confirm that it is the time-varying properties of real-time brain activity that are predictive of behavioral transitions, we repeated NNLS fitting on different extracted measures of neural activity including the instantaneous values, means, and SDs. The results illustrated in Figure S5 show that time-varying neural activity within each 10-s window (200 time points × 92 ROIs) and the region-resolved mean neural activity within a 10-s window (1 × 92 ROIs) can predict the larger effects of locomotion onset and offset but are unable to differentiate between initial and prolonged rest states. In contrast, the region-resolved SD of the signal within a 10-s window (1 × 92 ROIs), which captures the variance of each brain region, agreed well with correlation-based parameterization of all states, including initial and prolonged rest. This result is consistent with the signature of different behavioral states being related to dynamic brain activity patterns and not steady-state functional representations of the mouse’s physical behavior.

### Behavior-related changes in network dynamics are evident in real-time neural data

**Figure 5.**
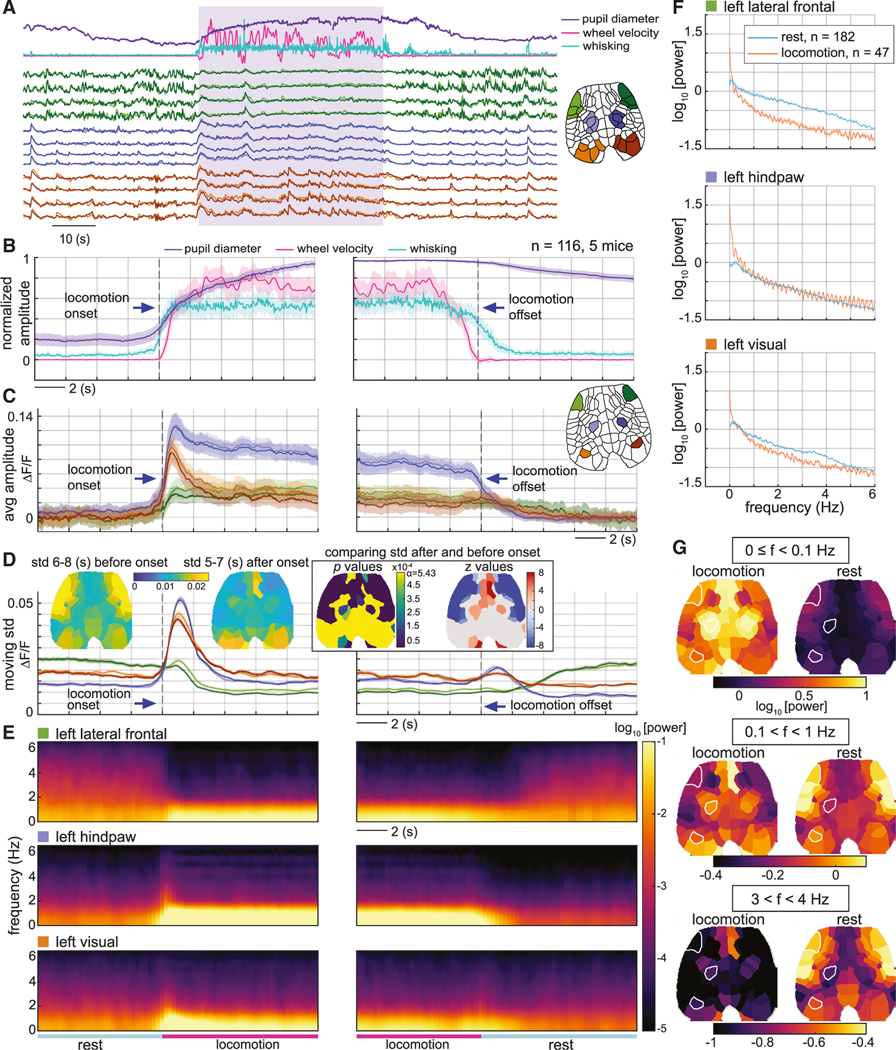
Dynamic properties of raw neural data during rest and locomotion transitions (A) Time courses of real-time behavior (top) and neural activity within the anterior lateral frontal cortex (green), hindpaw regions (blue), and visual cortex (red) for one example epoch. The locomotion period is indicated by the shaded area. (B-E) Averages across repeated locomotion bouts for (B) behavioral signals, (C) neural activity, (D) standard deviations (SDs) of neural activity overtime for prior 2-stemporal window, and (E) spectrogram of neural activity. Time courses were aligned around locomotion onset (left) and locomotion offset (right) (n = 116, five mice, mean ± SEM). Inset brain maps on (D) depict the SD of neural activity 6–8 s before and 5–7 s after locomotion onset, and the p values and z values of the comparison between the SDs (Wilcoxon rank-sum test, p < 0.05, Bonferroni corrected). *Z* scores set to zero for statistically insignificant comparisons. Note that sensory hindpaw and visual regions show a substantial increase in averaged moving-window SD during locomotion onset simply because signals are rapidly increasing on a single trial level. (F) Comparison of power spectra (mean ± SEM) of neural activity during locomotion (20 s in the middle of locomotion n = 47, 5 mice) and rest (30–50 s after locomotion, n = 182, five mice) for same 3 ROIs shown in (C). (G) Brain maps of the average spectral power for three different frequency bands comparing locomotion (left) with rest (right).

To illustrate how these behavioral correlation states are represented in raw neural activity data, Figure 5A plots real-time neural activity during the transition from rest into locomotion and back for a subset of anterior and posterior regions. Strongly varying activity in the frontal regions is seen during rest, which decreases during locomotion. This robust effect cannot be explained by movement artifacts or direct sensory or motor activation, as the activity patterns vary most strongly when the mouse is not actively engaged in locomotion and there is minimal motion.

Figure 5C plots the neural signal in three regions averaged over multiple locomotion onset and offset events (with behavioral parameters for the same epochs averaged in Figure 5B). As expected, strong neural activation in sensory hindpaw regions is seen during locomotion, as well as initial activation of the visual cortex, while only minimal net activation of anterior lateral frontal regions is observed.

In contrast, Figure 5D shows the temporal SD of neural signals in each region during a prior 2-s window (with insets mapping SDs for all regions 6–8 s before and 5–7 s after locomotion onset, with analysis of statistically significant changes). The anterior lateral frontal cortex exhibits high variance during rest, which begins to decrease more than 2 s before locomotion onset and drops significantly during locomotion, increasing again after locomotion offset. These patterns align with pupil diameter and whisking averages (Figure 5B), which can be seen to increase slightly before the onset of spontaneous locomotion, and remain elevated after locomotion ceases, consistent with expected arousal levels. Importantly, the presence of neural fluctuations causing this variance change is undetectable in the trial-averaged signals in Figure 5C.

Figure 5E shows corresponding average spectrograms of neural signals from the same sub-regions, while Figure 5F compares average power spectra for 20 s of locomotion vs. 20 s of rest. The spectral power of the anterior lateral frontal cortex in the frequency range of approximately 0.1–6 Hz during rest is higher than throughout the locomotion period, with a similar but smaller effect seen in the visual cortex for frequencies around 1–4 Hz. Figure 5G compares spatial maps of spectral power at different frequencies for locomotion and rest states, showing expected lower-frequency activity in sensory regions during locomotion but a shift to dominant higher-frequency frontal fluctuations during rest.

### Neural correlation changes between locomotion and rest also depict shifts in network activity

**Figure 6.**
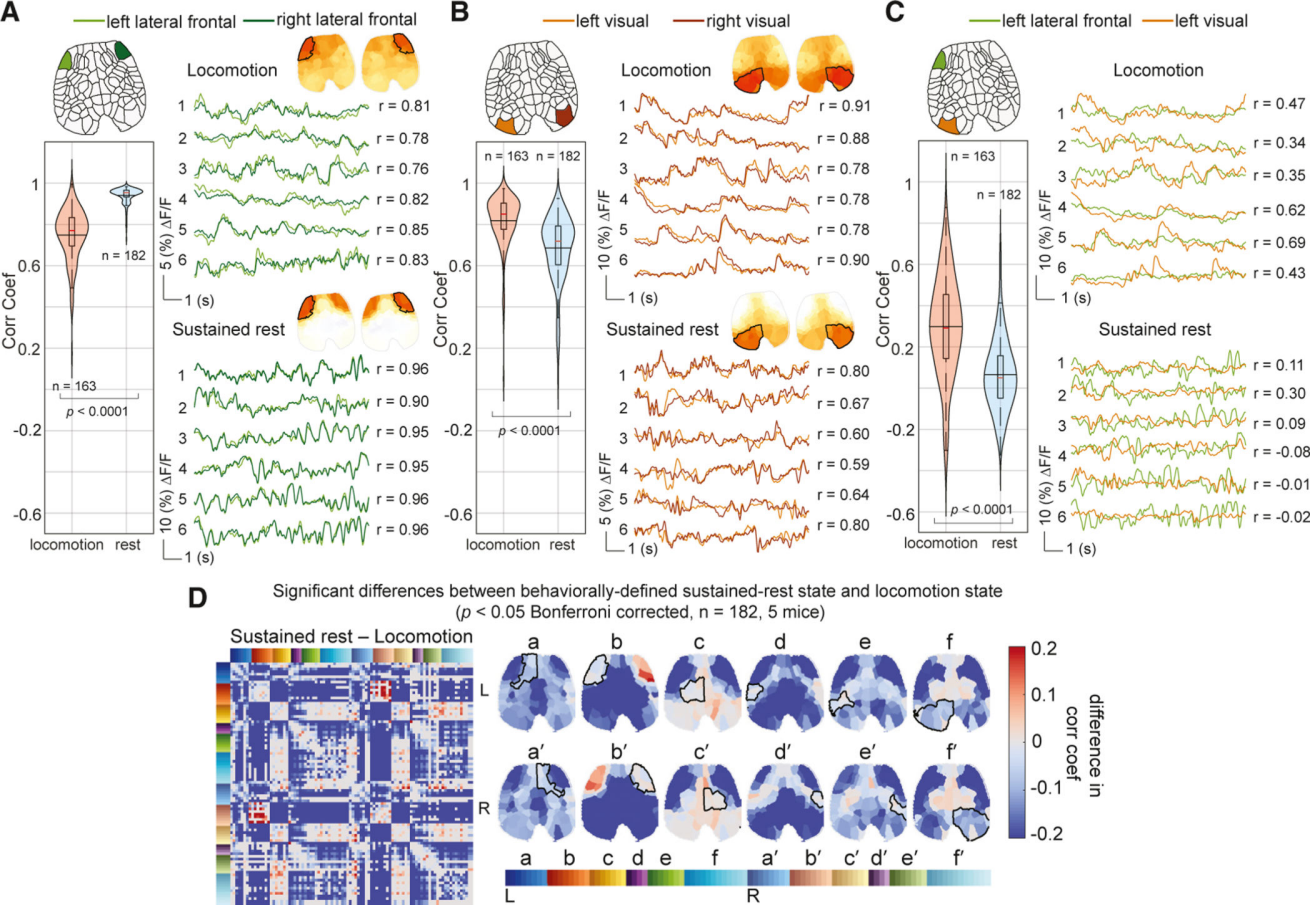
Comparing neural correlation shifts between locomotion and rest for different brain regions (A-C) Time series extracted from the ROIs indicated for six different epochs during locomotion (top) and sustained rest (below). (A) Bilateral correlation between anterior lateral frontal regions is significantly higher during rest. (B) Bilateral correlation between visual regions is significantly decreased during rest. (C) Anterior-posterior correlation is significantly decreased during rest. Across five mice, 163 locomotion and 182 resting bouts were compared (Wilcoxon rank-sum test, p < 0.05). (D) Differences in correlation maps between sustained rest and locomotion. Only statistically significant differences are displayed (Wilcoxon rank-sum test, p < 0.05, Bonferroni corrected, n = 182, five mice).

Although interregional correlations will be altered by functional representations of locomotion, and may even be affected by locomotion-related motion artifacts, here we explore how neural signals in different brain regions contribute to observed changes in correlation patterns between locomotion and rest states. We find that during rest compared with locomotion, (1) the anterior lateral frontal cortex becomes more bilaterally symmetric (Figure 6A, *Z* = 14.66, p < 1e–47, Wilcoxon rank-sum test), (2) the visual cortex becomes less bilaterally symmetric (Figure 6B, *Z* = −9.19, p < 1e–19), and (3) the anterior lateral frontal cortex becomes less synchronized with the posterior brain regions (Figure 6C, *Z* = −9.42, p < 1e–20). Figure 6D summarizes the significant differences in correlation between sustained-rest and locomotion correlation maps across all mice. We conclude that these effects, particularly anterior-posterior desynchronizations during rest, cannot be attributed to functional activation changes alone, and likely also reflect changes in dynamic brain network properties.

### Hemodynamic measurements recapitulate state dependence of neural activity correlations

The analysis above confirms that real-time neural dynamics in the awake, spontaneously behaving mice exhibit shifts in interregional correlation that relate to behavioral states as well as more subtle shifts in arousal. A major contributor to these changes in correlation is the modulation of highly variable patterns of neural activity in key brain regions, particularly anterior-posterior decor-relations during rest.

Our simultaneous acquisition of both neuronal activity and cortical hemodynamics affords the opportunity to explore how these patterns of neuronal correlation manifest in cortical hemodynamics, as a direct link to fMRI-based measurements of dynamic functional connectivity via the blood-oxygen-level-dependent (BOLD) signal.^15,24^

The above analysis sequence was thus repeated using simultaneously acquired measurements of changes in HbT concentration (Δ[HbT]=Δ[HbO]+Δ[HbR]) instead of neuronal activity. For simplicity, HbT was chosen over HbO or HbR as a more direct measurement of active hemodynamic modulation and, thus, neurovascular coupling.^14,15,25,26^ Unless otherwise noted, a 1.5-s lag was used^15^ to account for the anticipated delay of the hemodynamic response compared with neuronal activity, and hemodynamic data were temporally low-pass filtered at a cutoff frequency of 0.25 Hz to remove contamination from heart rate and breathing frequencies, as detailed in STAR Methods.

**Figure 7.**
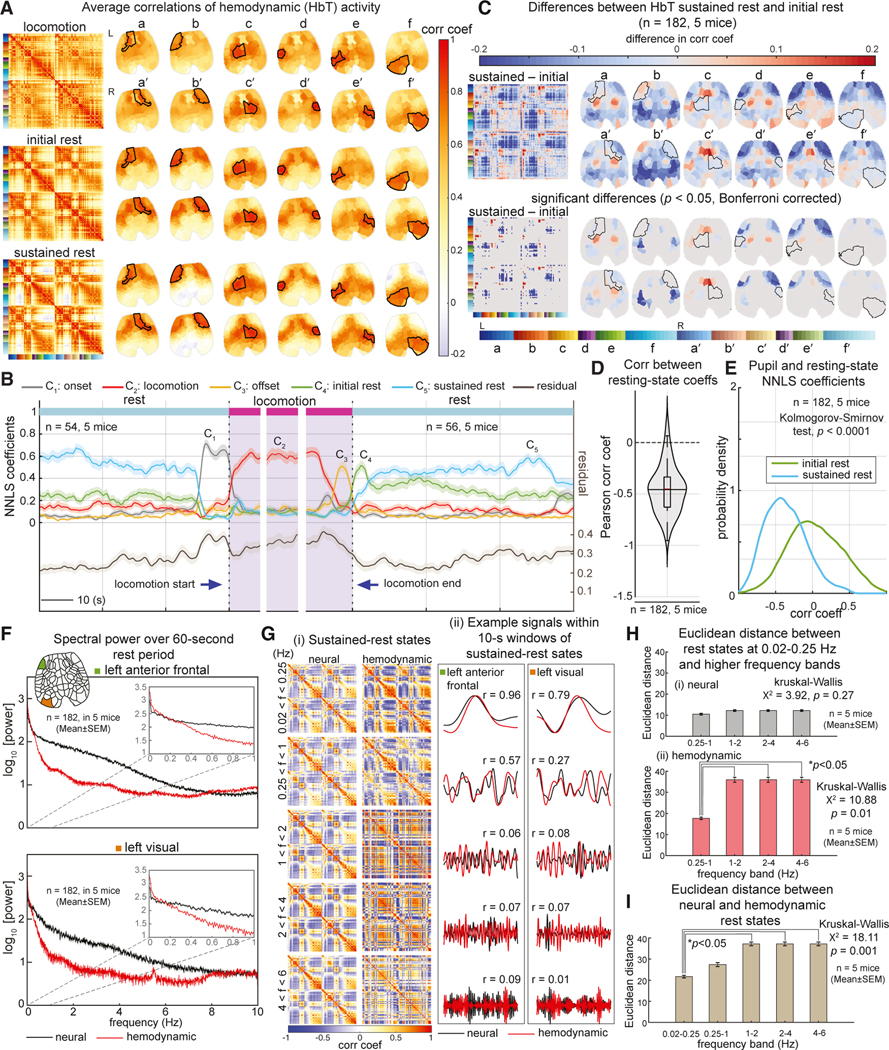
Analysis of hemodynamic behavioral correlation states and comparisons between hemodynamic and neural signal representations (A) Behaviorally defined locomotion and resting correlation states derived from hemodynamics(Δ[HbT]) forone example mouse, calculated similarlytothe neural states in Figure 3B. (B) Average coefficients predicted by NNLS models using hemodynamic states in (A) as a basis set (fitting to real-time hemodynamic 10-s moving-window correlation maps) across five mice (n = 54 [onset], 47 [locomotion], and 56 [offset] epochs), mean ± SEM. (C) Differences between sustained- and initial-rest states; (ii) includes only statistically significant differences (using a Wilcoxon rank-sum test, p < 0.05, Bonferroni corrected). (D) Negative correlation values between the initial-rest and sustained-rest NNLS coefficients. (E) Probability density of correlation values between pupil size and NNLS coefficients for initial and sustained rest calculated using kernel density estimation (p < 0.0001, Kolmogorov-Smirnov test). (F) Comparison of spectral power of neural and hemodynamic signals for the anterior frontal lateral (top) and posterior (visual, bottom) ROIs, over 60-s duration rest periods (n = 182, in five mice, mean ± SEM). (G) (i) Comparing neural (left) and hemodynamic (right) correlation maps for different frequency bands. Maps show correlations over 10-s windows during sustained-rest averaged over n = 63 epochs in one example mouse. All siganls were zero-mean adjusted and then temporally filtered over different frequency bands from low to high (top to bottom) (see STAR Methods). (ii) Time courses show example neural and hemodynamic signals temporally filtered over the same ranges as in (i) for one sustained-rest epoch extracted from the two ROIs indicated in (F). The r values show Pearson correlation coefficients between the neural and hemodynamic signals. (H) Euclidean distances between correlation maps for each frequency band in (G) relative to the 0.02–0.25 Hz frequency band for neural (top) and hemodynamic (bottom) measurements. (I) Euclidean distances between the neural and hemodynamic correlation maps at different frequency bands in (G). In (H) and (I), Kruskal-Wallis tests were used to compare groups (n = 5 mice, p < 0.05). See also Figures S6–S8 and Video S4.

Figure 7A shows the HbT-derived average behavioral correlation maps corresponding to locomotion, initial-rest, and sustained-rest states for one example mouse (the same mouse used in Figure 3B). In Figure S6, neural and hemodynamic behavioral correlation state maps are compared next to one another, demonstrating that the primary difference is a slightly broader spatial distribution of hemodynamic correlations, consistent with the spatial distribution of cortical vascular architecture. Additional behavioral state correlation maps for all five mice are presented in Figure S7, and real-time neural and hemodynamic moving window correlation maps are shown together with cortical activity and behavior in Video S4. Applying the same NNLS fitting approach as above using hemodynamic correlation maps, we found that the temporal evolution of hemodynamic brain states matches well with mouse behavior over all mice (Figure 7B).

As with neural activity, anterior-posterior decorrelation during rest is a prominent feature of the hemodynamic correlations. The raw differences in correlation maps between initial and sustained rest in Figure 7C clearly demonstrate this change, which was statistically significant. HbT-derived NNLS fit coefficients for initial and prolonged rest were moderately reciprocal over 182 resting-state epochs at least 60 s long across all mice (Pearson correlation coefficient mean = −0.5, SD = 0.22) (Figure 7D).

We also confirmed that the hemodynamic sustained-rest state was accompanied by a smaller pupil diameter, consistent with a lower arousal level. Figure 7E demonstrates the probability distributions of correlation values between the pupil size and the resting-state coefficients of the NNLS fit. These distributions were significantly different (using a Kolmogorov-Smirnov test, k = 0.26, p < 1e–4), which indicates that the hemodynamic initial and sustained rest manifest two distinct arousal levels.

These findings provide evidence that the correlation dynamics of hemodynamic data capture sufficient information to encode the same behavioral-state-related changes observable in the correlation structure of neural activity.

### Comparison of neural and hemodynamic correlations and activity dynamics

Understanding the spatiotemporal relationship between neural activity and changes in cortical hemodynamics, termed neurovascular coupling, is key to interpreting fMRI data. In Figures 7F–7I, we use our multimodal data to more closely examine the relationship between these two variables. Figure 7F compares the average spectral power of simultaneously acquired raw neural and hemodynamic signals for two example ROIs within the left anterior lateral frontal cortex and the visual cortex. Raw resting-state neural activity can be seen to span the full measurement range up to 10 Hz, which reflects our 20-Hz imaging rate, and the temporal properties of calcium indicators encoding neural activity.^16,27^ However, although the hemodynamic signal shows equivalent spectral power in lower frequency ranges, there is a clear cutoff at around <0.5 Hz, consistent with the temporal low-pass filtering effect of the cerebral vasculature.^15^ Figure S8A further confirms this relationship, showing the variance explained by hemodynamic data is well matched to neural data that have been temporally low-pass filtered at 0.25 Hz. Figure S8B directly compares hemodynamic time courses with raw, temporally low-pass filtered and time-shifted neural signals from bilateral regions within (i) the anterior lateral frontal cortex and (ii) the visual cortex, either side of locomotion offset. Consistent with prior work,^15^ (Figure 1) and additional examples shown in Figure S8C, neural events are clearly followed by increases in HbT signals in almost all regions, consistent with strong, positive neurovascular coupling. The relatively high correlation between hemodynamics and a temporally low-pass filtered and delayed version of the neural signal is mathematically consistent with a linear convolution of neural activity with a gamma distribution-shaped hemodynamic response function (HRF).^15,28^ However, we note that these results do not include comprehensive evaluation of the consistency of the cortical HRF across different brain regions.

To explore the frequency-dependence of both neural and hemodynamic correlation patterns, Figure 7G compares neural and hemodynamic behavioral correlation state maps for sustained rest, as well as example raw time courses temporally filtered over different frequency bands. Correlation maps match relatively well between neural and hemodynamic activity for lower frequencies (f < 1 Hz). However, when hemodynamic data are temporally high-pass filtered (f > 1 Hz), signals become dominated by correlated high-frequency breathing and heart-rate-dependent signals (visible at around 1–4 and 8–10 Hz, respectively in Figure 7F; note that these signals were excluded from earlier hemodynamic correlation analyses with a 0.25-Hz low-pass filter; see STAR Methods). These distorted hemodynamic correlation maps at higher frequencies underscore the temporal smoothing effect of neurovascular coupling, which removes higher-frequency information about neural activity from hemodynamic signals. Importantly, when neural data are high-pass filtered (4–6 Hz), interregional correlation patterns remain relatively matched to results obtained by low-pass filtering of both neural and hemodynamic data. These observations are quantified in Figures 7H and 7I by comparing the average Euclidean distances between each correlation map (see also Figures S8D and S8E for a comparison using correlations of correlation maps). This result is consistent with the broadband nature of spontaneous neural activity and preservation of correlation relationships between cortical regions across frequencies, as opposed to lower-frequency neural and hemodynamic patterns having a distinct source or relevance compared with high-frequency neural fluctuations.

## DISCUSSION

In this study, we examined the dynamic correlation structure of neural activity in the brain of awake, spontaneously behaving mice using simultaneous WFOM of both neuronal and hemodynamic activity. We observed readily interpretable neural activity patterns, especially relating to sensory regions during behaviors such as locomotion (Figures 1 and S2; Videos S1 and S2). However, neural activity also exhibited rich spatiotemporal dynamic structure across the cortex, even during quiescence or rest when mice were not actively engaged in activities such as grooming or locomotion (Video S2). The spatiotemporal properties of this resting activity delineated distinct and consistent cortical regions, revealing a data-driven functional topographic parcellation of the mouse cortex that adds detail to traditional anatomical atlases (Figures 2B and S3B).

Variations in the dynamic interregional correlation properties of this brain-wide activity were found to be predictive of both behavioral state and arousal level (Figures 3 and 4; and Video S4). In contrast, instantaneous neural signatures could not distinguish arousal-dependent shifts in brain states during rest (Figure S5). An increase in baseline neural fluctuations, predominantly in the anterior lateral frontal regions, was noted to be associated with decreased arousal level, resulting in anterior-posterior desynchronization during periods of quiescence (Figures 5 and 6).

Although hemodynamic representations of these correlation patterns were not identical to their neural counterparts, they clearly depicted differences between brain states and were predictive of behavior and arousal level (Figures 7A–7E). Analysis of the spatiotemporal relationship between neural activity and hemodynamics confirmed the preservation of low temporal frequency components of neural signals in hemodynamic recordings. Neural correlation properties were found to be consistent across the measured frequency range (0–10 Hz; Figures 7F–7I).

### Resting-state neural fluctuations vary with arousal level

One of the most prominent markers of sustained rest/low arousal that we identified was a strengthening correlation between the left and right anterior lateral frontal cortices, with a simultaneous desynchronization of both regions with posterior sensory areas such as the visual cortex. A major contributor to these correlation changes was the onset of strongly varying neural activity during rest.

Recent studies exploring the relationship between real-time cortical activity and behavior have noted that small spontaneous movements and whisking account for a modest portion of neural variance.^18,29^ However, the presence of spatiotemporally dynamic neural activity during quiet rest suggests that not all cortical neural activity relates linearly to overt behavior, providing a possible explanation for high residuals in these studies.

Our observations of dynamic resting neural activity, most strongly in the anterior lateral frontal, lateral somatosensory and visual cortices, are consistent with a range of other studies across modalities, species, and cortical areas. For example, wide-field neuronal GCaMP imaging has been used in mice^30^ to demonstrate that decreases in low-frequency power (3–6 Hz) in the primary visual cortex (V1), as well as frontal regions, are predictive of engagement in a visual task. In agreement with our results, this study recognized that task-engagement-related signals were not restricted to the cortical region engaged in the task. The suppression of rapid fluctuations in cortical neural activity by locomotion or movement has also been reported in the auditory cortex in mice^31^ and motor cortex in primates.^32,33^ Measuring intracellular membrane potentials, local field potentials (LFPs), and electroencephalography (EEG), a similar property was found in the mouse barrel cortex, where the resting-state fluctuations (1–5 Hz) were damped by active whisking.^34,35^ Vinck et al.^22^ recorded LFPs in mouse V1, finding that locomotion suppresses low-frequency LFP fluctuations (1–4 Hz), whereas stopping locomotion gradually increases low-frequency LFP power with a time course strongly correlated with pupil size. Interestingly, these higher-bandwidth electrophysiology measurements found a concomitant decrease in LFP gamma oscillations (55–66 Hz) during rest and an increase in these bands during locomotion. While using calcium indicators limited our ability to detect higher frequency shifts, our results suggest that a similar effect may be observed at higher frequencies across wider cortical regions.

We also observed that the anterior frontal cortex was less correlated with the posterior sensory regions during rest. If stronger synchronization, or functional connectivity between cortical brain regions can be considered as a measure of the information integration,^36^ then our findings may indicate reduced multisensory integration during periods of quiescence. This result could be consistent with EEG studies in humans that have shown that anesthesia decreases anterior-posterior synchronization and increases the bilateral coupling of the prefrontal cortex,^37^ a result recapitulated in rats using intracortical event-related potentials.^38^

In our study, some of the most prominent changes were observed in two bilateral regions of the anterior lateral frontal cortex (see Videos S1 and S2). The anatomical shape of these regions was highly consistent across animals and conditions and has been widely observed in previous wide-field calcium imaging studies.^18,39–43^ However, these anterior lateral frontal areas are not well represented in classical mouse brain atlases (see Figure S2), where they appear to bisect primary and secondary motor areas, or could potentially be defined as anterior lateral motor (ALM) cortex. One possible source of the strongly fluctuating neural activity during rest could be thalamocortical resonances or slow-wave sleep-type activity, enforced by an interaction between excitatory and inhibitory neurons in the thalamus.^44,45^ In combination with linear representations of sensory inputs and motor activity, these dynamic and overlapping oscillations could then contribute to changes in correlations and lead to apparent state-dependent dynamic changes in functional connectivity.

Synchronous fluctuations in neural activity across the brain are also important to recognize because they are likely a source of confusing and confounding variability for cellular-level measurements that are not on an ensemble scale (e.g., two-photon microscopy). In this context, such fluctuations would be regarded as task-independent noise. Experimental paradigms that average multiple repeated trials of stimuli or tasks will also average out these important signatures of brain state (Figure 5C), thereby overlooking their potentially important relationship to behavioral state, movement initiation and task performance.

### Hemodynamic coupling and relevance to human resting-state fMRI

We demonstrated that hemodynamic signals could differentiate between distinct behavior-driven states and identify switching between high and low arousal levels during rest. This result shows that, despite the relative complexity of the coupling between neural activity and brain hemodynamics, and the low temporal frequency content of the hemodynamic data, sufficient information about neural dynamics is retained in hemodynamic signals to depict similar correlation patterns and behavioral-state-dependent correlation dynamics. Our observation that high-frequency components of neural signals showed similar correlation patterns to low-frequency bands for both neural and hemodynamic signals is consistent with cross-frequency coupling across our 10-Hz measurement bandwidth. This finding suggests that hemodynamics (and thus the signals detected in resting-state fMRI) are providing information relevant to the properties of broadband spontaneous neural dynamics and do not just encode an independent source of low-frequency neural fluctuations.^46^ Meanwhile, the lack of correlation patterns in high-pass-filtered hemodynamic data underscores that the origin of dynamic correlation patterns and shifts in neural data cannot be due to residual hemodynamic cross-talk.

Widespread interest in resting-state fMRI stems from its potential to explore the human brain’s functional organization in both health and disease.^47^ Using rs-dFC, previous work has shown that spontaneous fluctuations in the BOLD signal (as a proxy for neural activity) are dynamically coherent.^3,5,48^ Although a growing body of research has been focusing on linking functional connectivity dynamics to mental processes, cognition, behavior, and disease,^10,49–53^ the origins and interpretation of ongoing time-varying synchrony patterns, and their relationships to disease states, have been the subject of significant debate.^54,55^ Our results demonstrate that dynamic changes in neuronal and hemodynamic correlation patterns can capture transitions in moment-to-moment brain states and arousal levels. A possible source of disease-related changes could thus be the sensitivity of resting-state fMRI to a patient’s physical and emotional response to the experience of undergoing an fMRI scan,^56^ which could include variations in alertness, anxiety, sleep transitions, mind wandering, restrained motion, or motor intents (analogous to the experience of a head-fixed mouse). It is therefore possible that disease-specific responses to the fMRI experience could underlie some of the predictive properties of dynamic functional connectivity transitions, particularly in psychiatric disease states. Conversely, several disease states, including glioma and acute stroke, have been shown to cause disturbances to both neurovascular coupling and the dynamic and correlation properties of resting-state neuronal activity in awake mice.^57,58^ In these cases, pathophysiological changes could more directly explain changes in the representations of these disease states in both task and resting-state fMRI assessments.

Overall, our results demonstrate that fluctuations in brain-wide neural and hemodynamic correlation patterns depict significant shifts and changes in the dynamic properties of brain activity (see Video S4). A significant component of these dynamics comes from widespread fluctuations in neuronal activity that increase during rest, but that are overlaid by functional representations of ongoing neuronal activity representing overt behaviors. A combination of these effects leads to dynamic changes in brain-wide correlations that vary during real-time behavior. Although these correlation patterns are typically referred to as changes in functional connectivity, our results suggest that they represent a more complex juxtaposition of transitions in the brain’s dynamic state. These insights into the contributions of different sources of neural variability to hemodynamic measurables may facilitate development of modes of fMRI rs-dFC analysis that can target and leverage the complex properties of these ongoing time-varying signatures and further elucidate their roles in relation to human brain disease.

### Limitations of the study

Although cortical hemodynamics were found to depict many features of neuronal correlations, hemodynamic correlation patterns were overall more variable across epochs than neural correlations, and residuals of our NNLS fits were higher for hemodynamic data. Higher noise levels and contamination of hemodynamic data with heart and breathing rate signals could have contributed to these effects. However, we also only explored coupling between Thy1-jRGECO1a-dependent neural activity and hemodynamics. Additional influences on hemodynamics could include coupling to other neural subtypes, including interneurons, and other origins of slow drifts or modulations of hemodynamics such as global brain modulations and systemic blood pressure and flow modulations.

Another important parameter in our analysis was our choice of a 10-s moving window over which to calculate our neural and hemodynamic Pearson correlations. This window size was chosen to capture transitions in behavioral states on a similar time frame to our relatively simple assessment of the mouse’s overt spontaneous behaviors and transitions in arousal, which occurred on the timescale of slow fluctuations in pupil diameter.^22^ This 10-s window also enabled us to evaluate inherently slower hemodynamic correlation patterns, enabling comparisons with the dynamics captured during resting-state fMRI. However, we acknowledge that the higher bandwidth of the neural and behavioral data acquired here could be used to evaluate transitions between more rapid and transient behavioral states that could be represented over correlation windows as short as 1–2 s. Such higher dimensional states likely encode detail about more rapid pupil fluctuations,^59,60^ more complex and non-overt behavior, whisking dynamics, and rapid transients in spontaneous neural activity (preparatory activity) that preceded locomotion onset. Our data suggest that continued exploration of neural dynamics and their relationship to brain state over a broad spectral range will likely provide further insights into brain-wide neural communication.

Future studies could leverage WFOM experiments (as well as datasets shared from this work) for development and testing of fMRI analysis methods, with neural data providing a ground-truth comparison. Studies could be extended to measurements of voltage, rather than calcium-sensitive indicators, and could record the activity of a range of neuronal (and even non-neuronal) cell types. These measurements could also permit finer dissection of the contributions of different cell types to correlation patterns and their relationships to complex behaviors across broad frequency ranges. Additional studies could incorporate specific stimuli and tasks to test the effects of external influences on brain dynamics, as well as optogenetic, pharmaceutical and disease-related perturbations to better characterize their effects on neuronal and hemodynamic signals, their correlation dynamics, and their relationships to behavior.

## STAR★METHODS

### RESOURCE AVAILABILITY

#### Lead contact

Further information and requests for reagents and resources should be directed to the lead contact, Elizabeth Hillman (elizabeth.hillman@columbia.edu).

#### Materials availability

This study did not generate new unique reagents.

#### Data and code availability

- Data generated and analyzed in this study (in the form of time courses extracted from regions of interest in brain imaging data, as well as extracted behavioral data) has been deposited and made accessible for analysis through Mendeley Data: http://dx.doi.org/10.17632/xd93nswg6h.1.
- Code used for the main preprocessing and analysis steps has been shared via GitHub: https://doi.org/10.5281/zenodo.7860561.
- Any additional information required to reanalyze the data reported in this paper is available from the lead contact upon request.

### EXPERIMENTAL MODEL AND SUBJECT DETAILS

#### Animals

Transgenic male adult Thy1-jRGECO1a mice expressing red-shifted calcium indicators were used in this study. The mice were bred from the line Tg(Thy1-jRGECO1a)GP8.20Dkim/J, purchased from the Jackson Laboratory. The mice were three months old when the experiments began. Mice were initially bred and housed in the animal facility at Columbia University. They were maintained up to five per cage with ad libitum access to food and water at a constant temperature (19–22°C) and humidity (40–50%) on a 12:12-hour light/dark cycle. After preparatory surgery, and between imaging sessions, animals were housed in individual cages within a satellite housing room close to the imaging system, maintaining consistent environmental conditions as detailed above. All animal procedures were reviewed and approved by the Institutional Animal Care and Use Committee at Columbia University in the City of New York (Protocol Number: AC-AAAS3453).

### METHOD DETAILS

#### Mouse preparation

To prepare each mouse for *in vivo* wide-field optical mapping (WFOM), a thinned-skull cranial window was created between the coronal and lambdoid sutures by anesthetizing mice using isoflurane, stabilizing the head in a stereotactic frame, retracting the scalp and gently thinning the skull to translucency with a dental burr. A thin layer of cyanoacrylate glue was then applied to the thinned skull to provide protection, reduce skull regrowth and improve transparency. A custom-made laser-cut acrylic head-plate was then glued to the edges of the skull to enable head fixation during imaging. Throughout this preparatory surgery body temperature was monitored and maintained using a heating pad and temperature probe. Mice were administered the analgesic buprenorphine for two days after surgery.

Longitudinal imaging measurements were acquired in a cohort of five age-matched mice. Acquisition started at least two days after surgery with at most 10 repeated 10-min recording sessions within one experimental session, up to eight experimental sessions spanning eight weeks. Before each recording, the cranial window was cleaned with deionized water, fully dried and covered with a glycerol mixture and glass coverslip to reduce specular reflections. After each experimental session, the headplate was cleaned, mice were removed from the imaging rig and returned to their home cage.

#### Monitoring locomotion, pupil and whisking

Two BFS-U3–16S2M-CS USB 3.1 Blackfly S Monochrome Cameras were used to monitor mouse behavior at a rate of 60 fps under infrared light illumination (850-nm wavelength with a HOYA R72 INFRARED filter). Both behavioral cameras were triggered to capture images at the same time as each WFOM image frame to ensure synchronization. One camera was widely angled to record the whole left side of the mouse’s body, and a mirror was placed below the wheel at a 45° angle to monitor the mouse’s paw movements. The other camera was set to closely capture the left side of the mouse’s face to record pupil and whisker movements. The open-source tracking software DeepLabCut^61^ was used to extract parameters from behavioral videos. Pupil size was tracked using eight circumferential points, fitting a circle to these eight points to estimate pupil diameter. To track whisking, nine points were used to annotate three visible whiskers (three points for each whisker) on the left side of the mouse’s face. Whisker speed was computed as the average frame-by-frame differences between the x-y coordinates of each whisker, calculated by Euclidean distance. The wheel’s rotary motion was recorded using a rotary encoder, and wheel movement velocity was computed as a locomotion measure. The time courses of pupil diameter, whisking speed and wheel velocity were used to quantify mouse behavior.

#### Wide-field optical imaging of neural activity and cortical hemodynamics

Data were acquired using a custom-made WFOM system, as depicted in Figure 1A Simultaneous recordings of the jRGECO1a fluorescence (neural) and reflectance (hemodynamic) data were made using an Andor Zyla sCMOS camera synchronized to three light emitting diodes (LEDs) strobing at 60 Hz (20 frames per second for each LED) with 4 × 4 binning (512 × 512 pixels per frame). The camera field of view was adjusted to capture the entire dorsal surface of the thinned cortex. Lime LED (565 nm: M565L3, Thorlabs; with 565/24-nm filter, Semrock BrightLine) light was used to excite jRGECO fluorescence. Reflectance signals were acquired during interleaved green (530 nm: M530L4, Thorlabs; with 530/43-nm filter, Semrock BrightLine) and red (625 nm: M625L4, Thorlabs; with 623/24 -nm filter, Semrock BrightLine) illumination. A dual-band emission filter (523/610-nm, Semrock BrightLine) was placed in the emission path to block lime excitation light but permit transmission of red jRGECO1a fluorescence and red and green reflectance.

### QUANTIFICATION AND STATISTICAL ANALYSIS

All data were analyzed using MATLAB (Mathworks). Statistical analyses were performed using non-parametric Wilcoxon rank-sum and Kruskal-Wallis tests, except in Figures S8D and S8E, where Fisher’s transformations of Pearson correlation coefficients were compared using one-way ANOVA and t-tests (p < 0.05). The multiple comparisons were performed with Bonferroni correction. The probability densities in Figures 4D and 7E were estimated by fitting a kernel distribution to the data using the Epanechnikov kernel function. Two-sample Kolmogorov-Smirnov tests were used to evaluate the significance of the difference between these probability distributions (p < 0.05). The average data are displayed as mean ± SEM, except for Figure S8A where the data are presented as mean ± SD. A total of 182 resting-state epochs were analyzed among five mice.

The boxplots of the violin plots (shown in Figures 4C, 6A–6C, and 7D) are centered on the median (the red horizontal lines) and extend to the 25th and 75th percentiles. The data points that are 1.5 times the interquartile range are considered to be outliers. The long horizontal black lines indicate the mean.

#### Hemodynamic conversion and JRGECO fluorescence correction

Reflectance recordings were used to estimate changes in concentrations of oxyhemoglobin (Δ[HbO]), deoxyhemoglobin (Δ[HbR]) and total hemoglobin (Δ[HbT]=Δ[HbO]+Δ[HbR]) using the modified Beer-Lambert law.^14^ JRGECO fluorescence recordings were further corrected for hemodynamic cross-talk and converted into ΔF/F, as detailed below.

The time-varying changes in cortical hemodynamics accompanying neural activity impose a varying absorption pattern on raw detected jRGECO1a fluorescence. Both the lime (565 nm) excitation light and the red (~630 nm) fluorescence light will experience attenuation from hemoglobin, and the wavelength-dependence of the absorption properties of oxy- and deoxy-hemoglobin requires a correction that considers both wavelengths. As derived below, we approximate this correction factor as a combination of the signals detected in our green and red reflectance data. The accuracy of this correction can be judged by observing how well it removes vessel-like artifacts in converted fluorescence data, as seen in Videos S1 and S3. We note that this correction is not wholly accurate for midline regions experiencing some strong motion-related fluctuations around the central sulcus, particularly during locomotion, so care was taken to ensure these signals did not impact the interpretation of our results. We also note that this correction differs from the correction of more common GCaMP fluorescence, which requires a more complex estimation/measurement of attenuation at blue excitation wavelengths.^14^

#### Derivation of hemodynamic correction factors for jRGECO1a fluorescence

Lime excitation light with intensity $I_{0,\text{ex}}$ enters the cortex and is scattered and attenuated along its path until reaching the fluorophore with intensity $I_{\text{ex}}$ where:

(Equation 1) $$I_{\text{ex}}\left(t,\lambda_{\text{ex}}\right)=I_{0,\text{ex}}\left(t,\lambda_{ex}\right){\text{e}}^{-\mu_{\text{a}}\left(t,\lambda_{\text{ex}}\right)X_{\text{ex}}\left(\lambda_{\text{ex}}\right)},$$

where $\mu_{a}$ is the wavelength- and time-dependent absorption coefficient experienced by the excitation photons, and $X_{ex}$ represents the (wavelength-dependent) distance traveled by the photons (their pathlength). This incident light is then converted to fluorescence emission light by the fluorophore, becoming

(Equation 2) $$I_{0,\text{em}}\left(t,\lambda_{\text{em}}\right)=c_{F}F(t)I_{\text{ex}}\left(t,\lambda_{\text{ex}}\right),$$

where $c_{F}$ is the fluorophore concentration and F(t) represents the time-varying intracellular calcium concentration of the jRGECOIa expressing neurons (our measure of neuronal activity). This emitted red fluorescence light must then reach the surface of the cortex, experiencing wavelength-dependent scattering and absorption, and emerging with intensity:

(Equation 3) $$I_{\text{em}}\left(t,\lambda_{\text{em}}\right)=I_{0,\text{em}}\left(t,\lambda_{\text{em}}\right)e^{-\mu_{\text{a}}\left(t,\lambda_{\text{em}}\right)X_{\text{em}}\left(\lambda_{\text{em}}\right)}.$$

Combining these 3 equations, we see:

(Equation 4) $$I_{\text{em}}\left(t,\lambda_{\text{em}}\right)=c_{F}F(t)I_{0,\text{ex}}\left(t,\lambda_{\text{ex}}\right)e^{-\mu_{\text{a}}\left(t,\lambda_{\text{em}}\right)X_{\text{em}}\left(\lambda_{\text{em}}\right)}e^{-\mu_{a}\left(t,\lambda_{\text{ex}}\right)X_{\text{ex}}\left(\lambda_{\text{ex}}\right)}.$$

If we divide this detected signal by a baseline signal (e.g., the average of 100 image frames), we cancel out the temporally constant initial intensity term $I_{0,\text{ex}}\left(t,\lambda_{\text{ex}}\right)$ (accounting for spatially uneven illumination) and the spatially dependent fluorophore concentration $c_{F}$ yielding:

(Equation 5) $$Inorm_{\text{em}}\left(t,\lambda_{\text{em}}\right)=\frac{I_{\text{em}}\left(t,\lambda_{\text{em}}\right)}{l_{\text{em}}\left(\text{base},\lambda_{\text{em}}\right)}=\frac{F(t)}{F(\text{base})}e^{-\text{Δ}\mu_{\text{a}}\left(t,\lambda_{\text{em}}\right)X_{\text{em}}\left(\lambda_{\text{em}}\right)}e^{-\text{Δ}\mu_{\text{a}}\left(t,\lambda_{\text{ex}}\right)X_{\text{ex}}\left(\lambda_{\text{ex}}\right)},$$

where $\text{Δ}\mu_{a}$ is now the change in absorption coefficient relative to the baseline state. If we now consider the form of our reflectance signals, each detected photon enters the tissue with intensity $I_{0,\text{R}}\left(t,\lambda_{R}\right)$, is scattered and absorbed and exits the cortex with intensity:

(Equation 6) $$I_{R}\left(t,\lambda_{R}\right)=I_{0,R}\left(t,\lambda_{R}\right)e^{-\mu_{a}\left(t,\lambda_{R}\right)x_{R}\left(\lambda_{R}\right)}.$$

Dividing by the baseline reflectance image, we similarly get:

(Equation 7) $$Inorm_{R}\left(t,\lambda_{R}\right)=\frac{I_{R}\left(t,\lambda_{R}\right)}{I_{R}\left(\text{base},\lambda_{R}\right)}=e^{-\text{Δ}\mu_{a}\left(t,\lambda_{R}\right)X_{R}\left(\lambda_{R}\right)}.$$

Here, it is important to consider that $X_{R}$ is likely to be a longer path than that traveled by a fluorescence photon, which we assume will interact with a fluorophore at some point along its path; however, it is also important to note that pathlength estimates for scattered light are only average approximations and can depend on many factors specific to the tissue-see.^14^ If we assume that our red reflectance experiences a similar absorption coefficient as our red fluorescence emission, and our green reflectance experiences a similar absorption coefficient as our lime fluorescence excitation, then the reflectance measurements can be used to approximate the time-varying absorption component of Equation 4 such that:

(Equation 8) $$\frac{Inorm_{\text{em}}\left(t,\lambda_{\text{em}}\right)}{Inorm_{R}{\left(t,\lambda_{\text{red}}\right)}^{Pr}Inorm_{R}{\left(t,\lambda_{\text{green}}\right)}^{Pg}}=\frac{F(t)}{F(\text{base})}.$$

Producing a measurement proportional to the percentage change in jRGECOIa fluorescence. The unknowns in this equation are Pr and Pg, which represent the ratio of the distances traveled by lime excitation light vs. green reflectance, and red emission vs. red reflectance photons, respectively, such that: $Pg=X_{ex}\left(\lambda_{ex}\right)/X_{R}\left(\lambda_{\text{green}}\right)$, $\mathrm{Pr}=X_{em}\left(\lambda_{em}\right)/X_{R}\left(\lambda_{red}\right)$. These coefficients were estimated using an iterative procedure that compared the vascular features of images after correction, with final values used for all data and all mice of Pg=0.2−0.3 and Pr=1.1−1.8. In order to calculate the baseline signal for both fluorescence and reflectance data, 100 image frames were averaged during a period of rest (selected as frames in which the wheel rotary movement did not exceed a predetermined level).

#### WFOM data temporal filtering, denoising and time course extraction

For raw data time courses and images shown in Figures 1, S2, Videos S1 and S2, heart rate-related fluctuations were reduced by dividing images by a spatially uniform, temporally high-pass Altered (>1Hz for hemodynamic and >2 Hz for neural) global mean component for each LED channel. Data were then denoised using principal component analysis of each 10-minute-long recording, retaining the first 200 spatiotemporal components for raw red and green reflectance (hemodynamic) data, and 300 components for raw lime (neural) data (cut-offs determined using the examination of spatiotemporal noise characteristics of each component and the elbow point of the variance explained). Signals then underwent hemodynamic correction and conversion to [Hb]. Time courses were extracted from the regions of interest indicated. Hemodynamic time-series data in Figure S2 and Videos S1 and S3 underwent 3-point (0.15 s) temporal smoothing.

For all correlation analysis, time courses were extracted from regions of interest determined through parcellation for each mouse (detailed below), spatially registered for each recording session, and taken as the average red, green and lime signal within each ROI. Unless otherwise stated, the only pre-processing of these raw WFOM reflectance and fluorescence signals for correlation analysis was temporal low-pass filtering with a cutoff frequency of 6.5 Hz to reduce global heart-rate-dependent signals followed by hemodynamic correction and conversion to [Hb]. The frequency analysis of neural and hemodynamic signals revealed breathing vascular artifacts around the frequency range of 1–4 Hz that were prominent in the hemodynamic reflectance measurements, but not fluorescence. To avoid spurious correlations, hemodynamic data were further low-pass filtered at 0.25 Hz. Filtering procedures were performed using zero-phase filters (filtfilt) in MATLAB (Mathworks) with a filter order of 20 and a pass-band ripple of 0.2. These data were used in Figures 2, 7, S3, and S8.

In Figures 7 and S3, the raw data were used to analyze and visualize the frequency information of neural and hemodynamic activity over the 10 Hz measurement bandwidth. For the purpose of comparing resting-state correlation maps across various frequency bands in Figure 7B, the neural and hemodynamic signals were first transformed to have zero means. Using Fast Fourier Transform (FFT), the signals were then filtered with multiple band-pass frequency ranges.

#### Region of interest parcellation of neural data

To generate our cortical topography, k-means clustering was run with a wide range of parameters and cluster numbers across many mice, sessions and epochs. The general spatial structure of parcellation results was found to be widely consistent, although the most detailed and clear delineation was found when clustering data during periods of quiet rest (cortical topography clustering results using data acquired during periods of vigorous locomotion tend to coalesce highly active areas such as a hindpaw and forepaw sensorimotor regions, while this locomotion state also suppresses fine patterns of variance in other brain regions as examined further in Figure 5).

To compare clustering results and determine the optimal number of clusters, we used non-negative least squares fitting (NNLS) where temporal centroids for each clustering result were used as a basis set $\text{H}{\left(\text{t}\right)}_{\text{n}}$ and neural imaging data M(x,y,t) were represented as:

(Equation 9) $$M(x,y,t)=\sum_{n=1}^{N}W{\left(x,y\right)}_{n}H{\left(t\right)}_{n}.$$

NNLS generates spatial representations $\text{W}{\left(\text{x},\text{y}\right)}_{\text{n}}$ as non-integer coefficients indicating how much of each time course $\text{H}{\left(\text{t}\right)}_{\text{n}}$ is present within each image pixel. This formulation permits analysis of the spatiotemporal residual of the linear fit, and thus the goodness of fit for different numbers of cluster components N. These results were also compared to principal component analysis (PCA) of the same neural data to determine the overall dimensionality of the data and the threshold number of components providing high explained variance. Results are shown in Figure S3B, which demonstrates that 92 is a suitable threshold for the number of cluster components, with only small reductions in residual and improvements to the variance explained when increasing clusters from 92 to 200 or more.

To generate a generalized parcellation that would permit consistent comparisons across all mice, we then performed the following procedure. K-means clustering with 100 replicates, correlation distance metric and 46 clusters was applied to one hemisphere of data during a 60-s rest period from one mouse. The resulting spatial clusters (representing an integer form of $\text{W}{\left(\text{x},\text{y}\right)}_{\text{n}}$ as detailed above), were then reflected onto the contralateral cortex and signals extracted from each region of interest were used as an initialization condition for k-means clustering on the contralateral side data. Temporal correlations between resulting cluster centroids on the left and right sides of the cortex were used to establish bilateral pairs of regions, and thus the paired sequence order of cluster ROIs on the left and right sides of the cortex.

Once this bilateral, 92 component map was established for one mouse, it was registered onto the brains of other mice using an affine transform. As before, these ROIs were used in each mouse to extract centroid time courses for rest epochs, which were used as the initial condition for repeated clustering to refine the ROI map for each specific mouse (while constraining the general position and order of centroids between mice). The most commonly occurring mode map for these clustering steps was chosen as the final map for each mouse.

Each mouse-specific ROI map was registered onto the brain image of each mouse in each session and used to extract time-series data as detailed above. To reduce the effects of small motion artifacts, averaged signals were extracted from each of these 92 ROIs from raw data (red and green reflectance, and raw fluorescence data). These signals were then converted to Δ[HbO] and Δ[HbR] and Δ[HbT], as well as application of the hemodynamic correction detailed above to fluorescence measurements to calculate $\text{ΔF}/{\text{F}}_{0}$. This approach was cross-validated with signals extracted from data that was converted to Δ[Hb] and ΔF/F in a pixel-wise manner (as shown in Videos S1, S2, and S3).

#### Calculation of correlation maps and cortical visualizations

Unless otherwise noted, the pairwise Pearson correlation coefficients between time courses for the 92 ROIs were calculated over 10-s temporal windows, resulting in 92 × 92 correlation maps. Before computing correlation matrices, the first-degree polynomial trends in windowed hemodynamic data were removed.

To facilitate visual comparison of the correlation maps, six subgroups of ROIs (a-f) within each hemisphere were found using the *k*-means algorithm applied to the correlation map data (see Figure 2C). These groupings were used to arrange the order of all 92 × 92 correlation maps for each mouse, spanning sub-regions in a-f (left) and a’-f’ (right).

Although data in a full 92×92 correlation map can be visualized as 92 sequential cortical maps (as shown in Video S3), we noted that the 12 clusters a-f’ found above formed contiguous sub-regions on the cortical map (Figure 2D). To enable simpler visualization, we converted each 92 × 92 correlation map into a 12 × 92 element map by averaging correlation values within each of these 12 clusters. Projecting each of the 1 × 92 rows of the resulting correlation map onto the cortical surface produces the 12 representations shown in (Figure 2E), which approximate the correlation of each of the larger ROIs (a-f, a’-f’) with all of the other 92 regions of the cortex. The same approach was used for all subsequent figures (Figures 3B, 4E, 6D, 7A, 7C, S5, and S6), providing a simplified view of bilateral correlation patterns across the brain for different conditions and behavioral states.

#### Defining behavior-driven correlation states

The first step of our linear modeling analysis (Figures 3, 7, and S5) used information from the wheel rotary encoder to extract locomotion bouts along with the periods of quiescence before and after locomotion from each recording session.

Behavior-driven correlation states were then computed for each mouse. The locomotion-onset state was calculated as the average of the correlation maps over the temporal windows overlapping with 5-s rest and 5-s locomotion, using locomotion bouts with at least 10-s duration and 60-s pre-locomotion rest. The sustained locomotion state was calculated as the average correlation map over 10-s periods right in the middle of each locomotion bout, using locomotion bouts with at least 20-s duration. The locomotion-offset state was calculated as the average of correlation maps over temporal windows with 5-s locomotion and 5-s rest, using bouts with at least 20-s locomotion duration and 10-s post-locomotion rest. The initial rest state was calculated as the average correlation maps over the first 10s immediately after locomotion cessation (stationary wheel) for locomotion bouts lasting at least 5 s, with at least 60 s post-locomotion rest. The sustained rest state was calculated as the average correlation map over 10 s, starting 40 s after locomotion cessation for the same bouts as for the initial rest state.

The 40 s delay chosen for the sustained rest state was based on prior work^22^ that found that pupil size, on average, returns to baseline around 40 s after mice stop running. Our results are consistent with this observation as illustrated in Figure 4A.

#### Linear model of behavior-driven correlation states

To determine whether the average behavioral correlation states derived as detailed above are predictive of real-time mouse behavior, a non-negative least squares (NNLS) fit was performed using these five correlation states $\text{x}{\left(\text{r},\text{r}\right)}_{1:5}$ as a basis set (where r = 1 to 92 regions of interest). Every real-time correlation map d(r,r,t) (using a moving 10-s window) throughout the recording sessions was modeled as a linear combination of the basis set, solving for $\text{c}{\left(\text{t}\right)}_{1:5}$, the time-varying coefficient represents the contribution of each basis state to the each real-time correlation map:

(Equation 10) $$d(r,r,t)=\sum_{n=1}^{5}x{\left(r,r\right)}_{n}c{\left(t\right)}_{n}.$$

The goodness of fit between each real-time correlation map and the model prediction was evaluated as $\frac{{\left\|{\left(d-cx\right)}^{2}\right\|}_{1}}{{\left\|d^{2}\right\|}_{1}}$. To calculate the average coefficients estimated by the predictive model, locomotion bouts were sorted either based on locomotion-onset time or locomotion-offset time. Locomotion periods were aligned 5 s after locomotion onset. Our NNLS results in Figures 3 and 7 were calculated using 5-fold cross-validation. As such, we ensured that the NNLS model was applied to frames that were not used to calculate the basis sets.

## Supplementary Material

1

2

3

4

5

## Figures and Tables

**Table:** KEY RESOURCES TABLE

REAGENT or RESOURCE	SOURCE	IDENTIFIER
Deposited data		

WFOM and behavioral datasets	This study	Zenodo: https://doi.org/10.S281/zenodo.7968402

Experimental models: Organisms/strains		

Tg(Thy1-jRGECO1a)GP8.20Dkim/J	Jackson Laboratory	RRID:IMSR_JAX:030525

Software and algorithms		

MATLAB 2016a–2019b	MathWorks	https://www.mathworks.com/products/matlab.html
Code used for the main data processing and analysis steps	This study	GitHub: https://doi.org/10.5281/zenodo.7860561

## Highlights

- Interregional neural correlations reveal a compelling functional cortical topography
- Ongoing neural activity shows time-varying correlation structure across the cortex
- Neural activity correlation patterns can predict behavioral states and arousal levels
- Brain hemodynamics recapitulate the dynamics of neuronal correlation patterns
