# Supplementary material for: Cortex-wide neural dynamics predict behavioral states and provide a neural basis for resting-state dynamic functional connectivity
Source: Cell Rep. Author manuscript; Available in PMC 2023 Oct 23. (PMC10592480; doi:10.1016/j.celrep.2023.112527)
Supplement: 1 [file NIHMS1912759-supplement-1.pdf]

Cell Reports, Volume *42*

Supplemental information

Cortex-wide neural dynamics predict behavioral states and provide a neural basis for resting-state dynamic functional connectivity

Somayeh Shahsavarani, David N. Thibodeaux, Weihao Xu, Sharon H. Kim, Fatema Lodgher, Chinwendu Nwokeabia, Morgan Cambareri, Alexis J. Yagielski, Hanzhi T. Zhao, Daniel A. Handwerker, Javier Gonzalez-Castillo, Peter A. Bandettini, and Elizabeth M.C. Hillman

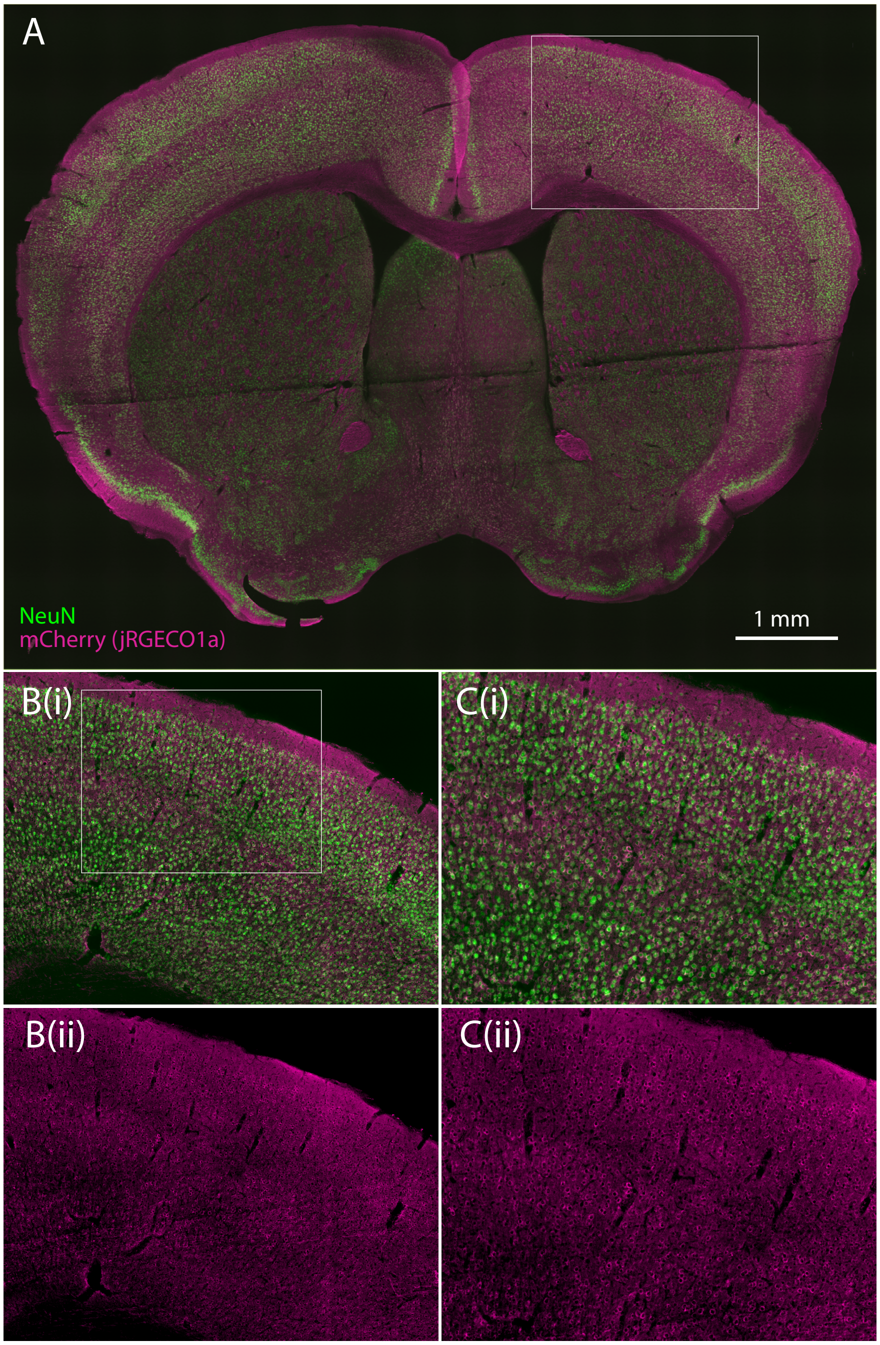

Supplemental Figure S1. Immunohistochemistry of a representative Thy1-jRGECO1a mouse. Related to **Figure 1** and STAR methods. Mouse line GP8.20Dkim/J, staining for mCherry (jRGECO1a, magenta) and NeuN (green). Acquired at 10x magnification using spinning disk confocal microscopy. **(A)** Shows a tiled image of the whole coronal section with magenta and green labels overlaid. **(B(i))** shows the zoomed-in region indicated in **(A),** with **(B(ii))** showing only the magenta (jRGECO1a) channel. **(C(i))** shows the zoomed-in region indicated in **B(i)**, with **C(ii)** showing only the magenta (jRGECO1a) channel. Images confirm that Thy1-jRGECO1a is expressed in layers 2/3 and 4 and is colocalized with neuronal marker NeuN.

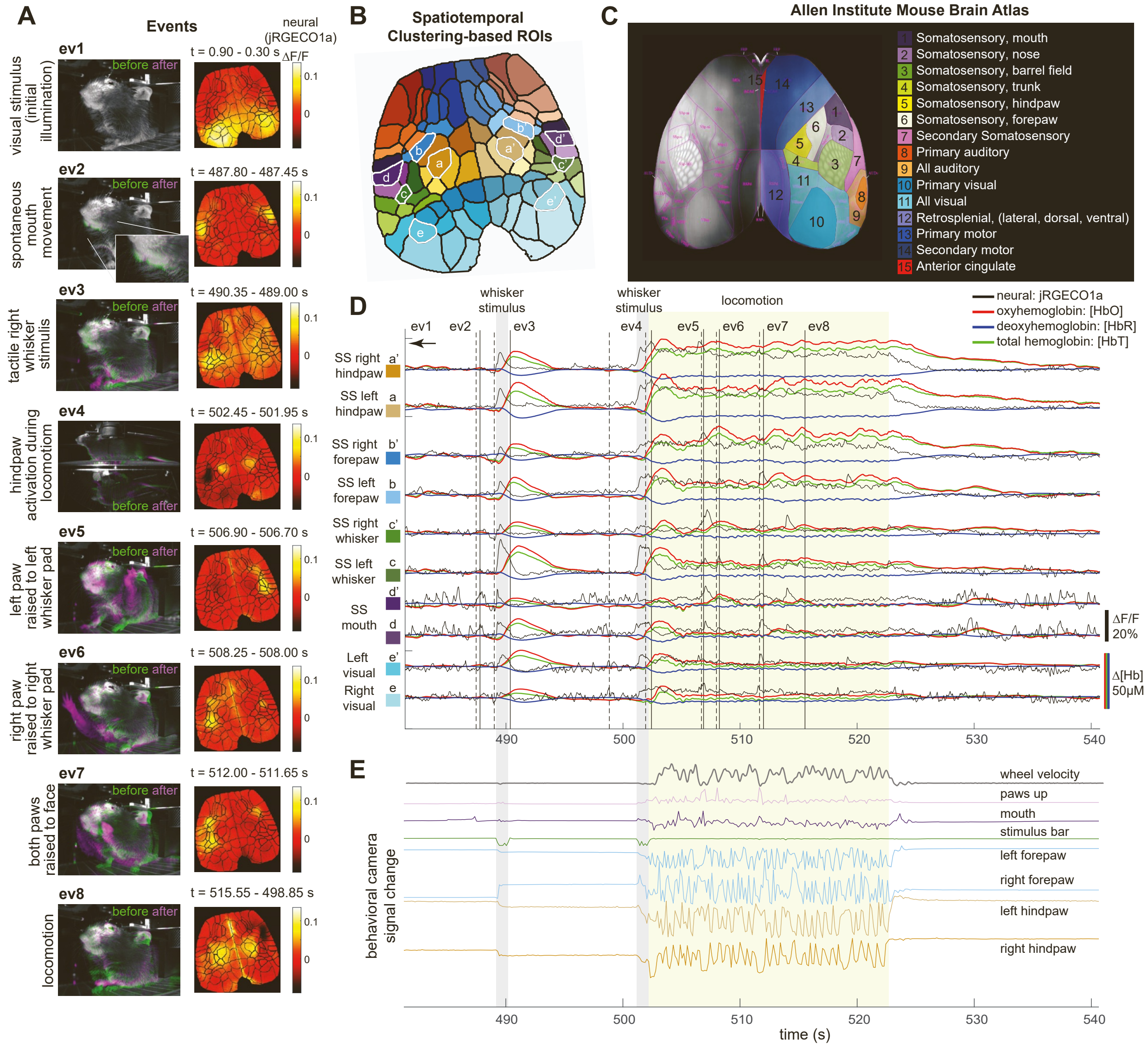

Supplemental Figure S2. Comparison of functional and anatomical cortical topography. Related to **Figure 1**. Data are shown for an epoch where the mouse received several randomized tactile whisker stimuli. For the first stimulus, the mouse reacted but did not run; however, the mouse ran and touched its face due to the second stimulus. **(A)** Specific events during the 60-second epoch, with camera images shown on the left and neural cortical images (corrected jRGECO1a) maps on the right. Camera images show a merge of an earlier (green) and later (magenta) frame depicting the nature of the event. Neural activity maps similarly subtract an earlier from a later frame (with a ~ 0.5 s delay after the camera image) showing the cortical representation of the event. The timing of each event frame (solid line) and reference frame (dotted line) are indicated in **D**. Neural data images are overlaid with the mouse-specific cortical parcellation generated through spatiotemporal clustering, showing that regions delineated clearly overlay functional brain regions corresponding to each event, from top to bottom: visual cortex at the onset of acquisition, sensory mouth during a mouth movement, sensory whisker after stimulation, sensory hindpaw during locomotion onset, left sensory forepaw and whisker, right sensory forepaw and whisker, both left and right sensory forepaw and right whisker during face touching, and left and right forepaw and hindpaw during locomotion. **(B)** An enlarged version of the cortical topography highlighting putative functional regions a-e and a'-e' labeled bilaterally, with their time courses for both neural activity and hemodynamics plotted in **D**, where SS denotes somatosensory cortex. **(C)** A reproduced version of the Allen Brain Atlas indicating anatomically derived sensory and motor regions [S1]. We note the reasonable correspondence between the locations of sensory regions in our functionally derived topographic map, although note that representations of regions in the frontal motor cortex are inconsistent between the topographies, in particular, the lack of delineation of 'anterior lateral' regions in the Allen atlas, as detailed further in the main paper. **(D)** Consistent with data in the main paper, time courses show good representations of the mouse's real-time behavior including flinching during the first whisker stimulation, strong hindpaw, forepaw and visual sensory activity during locomotion, particularly at the onset, and variable spontaneous activity in visual and anterior sensory regions (e.g., mouth) during rest. Clear neurovascular coupling relationships between neural and HbT, HbO and HbR can be seen. Gray shading indicates delivery of tactile whisker stimulus, yellow shading indicates a bout of locomotion **(E)** Behavioral parameters extracted from the video recording, showing temporal variations of the indicated body parts and the horizontal wheel movement velocity.

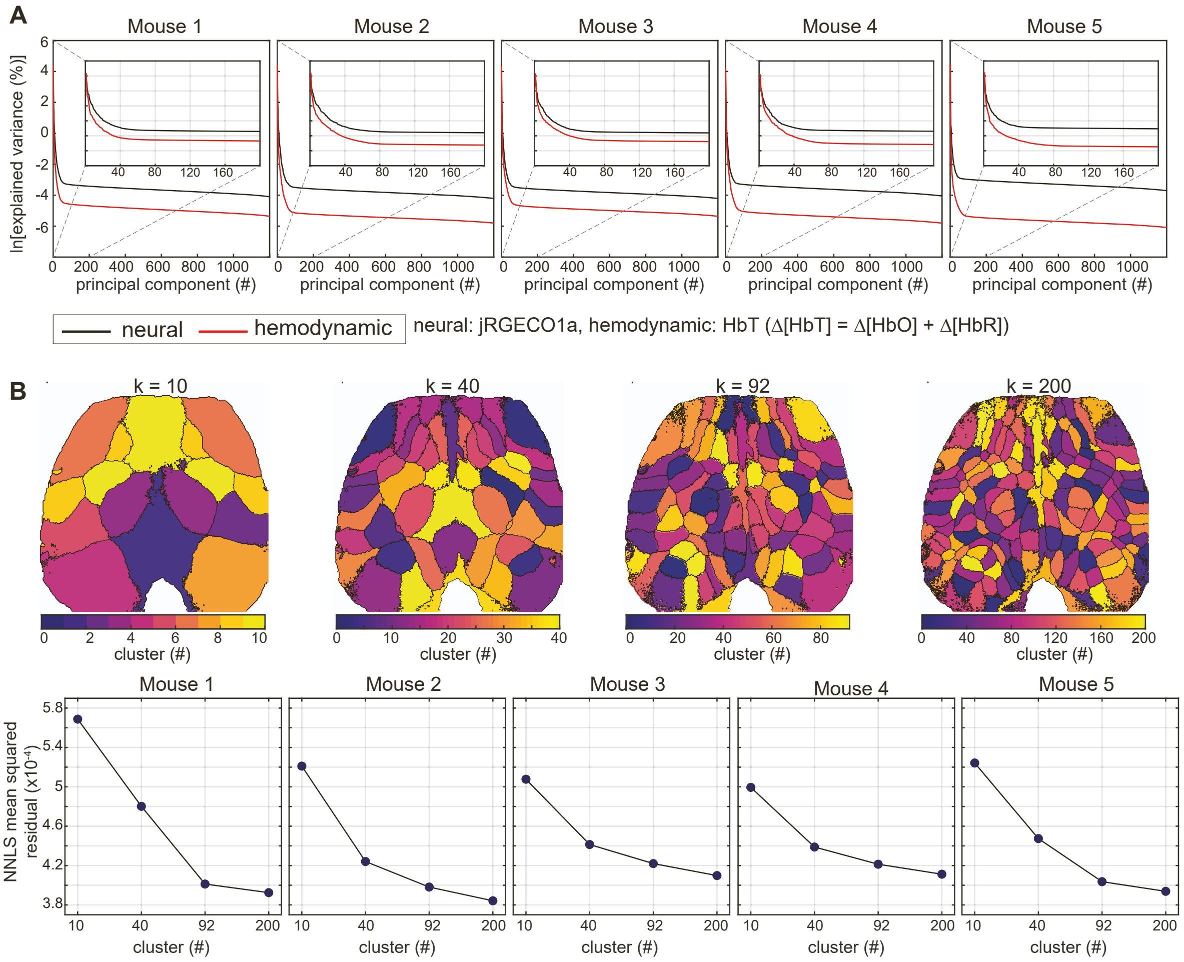

Supplemental Figure S3. Explained variance of neural and hemodynamic data. Related to **Figure 2**. **(A)** Natural logarithm of explained variance applying principal component analysis on both neural (black) and hemodynamic (red) activity. This demonstrates the dimensionality of one example of 60-second resting-state raw data for each mouse. **(B)** Comparing brain parcellation with different numbers of ROIs using raw neural data. The brain maps depict the output of the k-means clustering on one 60-second resting-state epoch. To compare clustering outputs, the residual between the raw data and the data reconstructed by a non-negative least squares (NNLS) model using the clusters as the basis was computed. For each mouse, the plots show that the residual decreases as the number of ROIs increases.

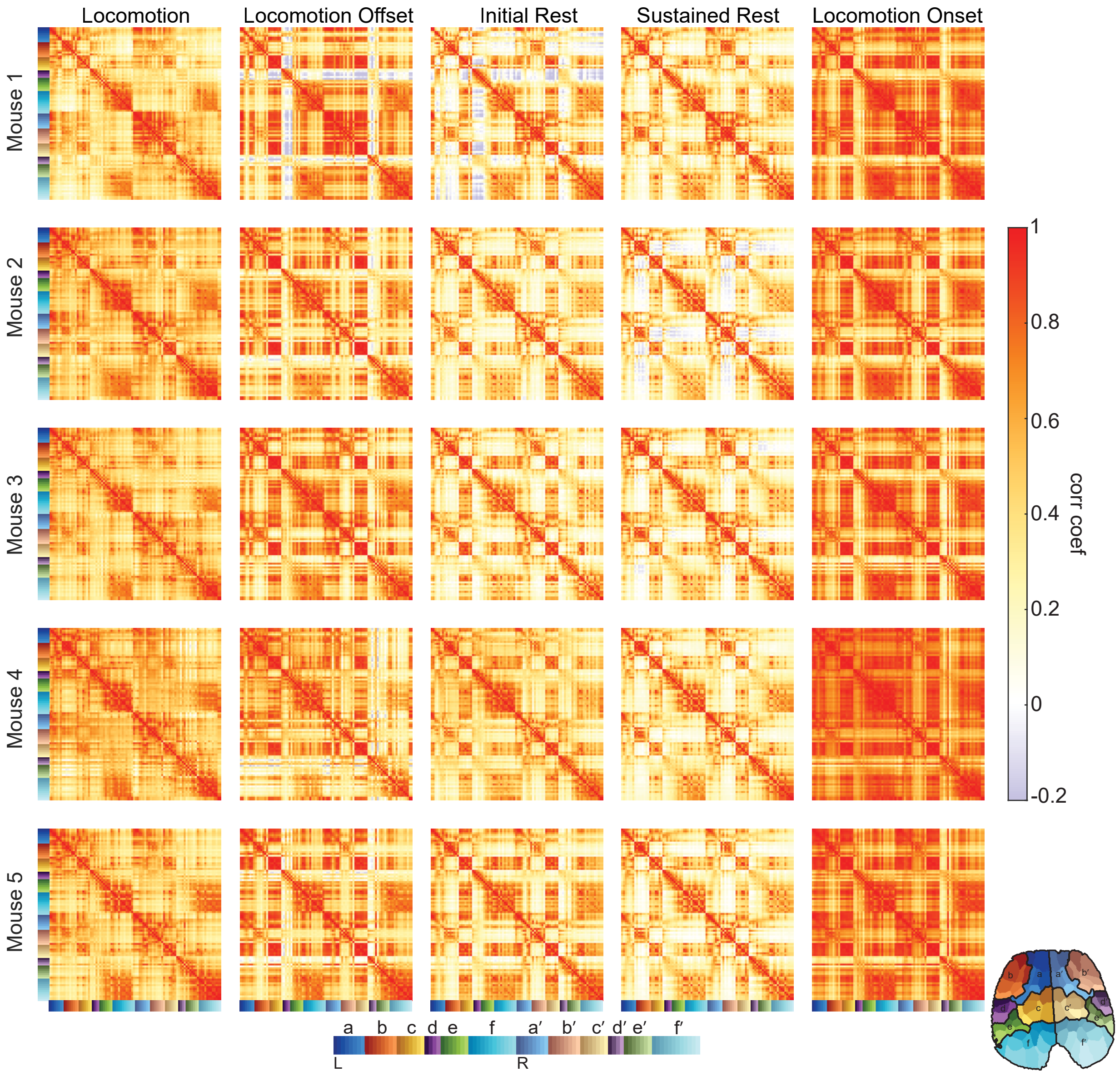

Supplemental Figure S4. Behaviorally-defined neural brain states for all five mice. Related to **Figure 3.** Neural signals were time-locked to five distinct behavior windows lasting ten seconds each, as shown in **Figure 3A**: locomotion, locomotion offset, initial rest (immediately after locomotion end), sustained rest (40-s rest after locomotion end), and locomotion onset. The correlation maps were computed as pairwise Pearson correlation coefficients between the neural signals of 92 ROIs within each window. The brain states were defined as the average of the correlation maps across all epochs within each behavior window.

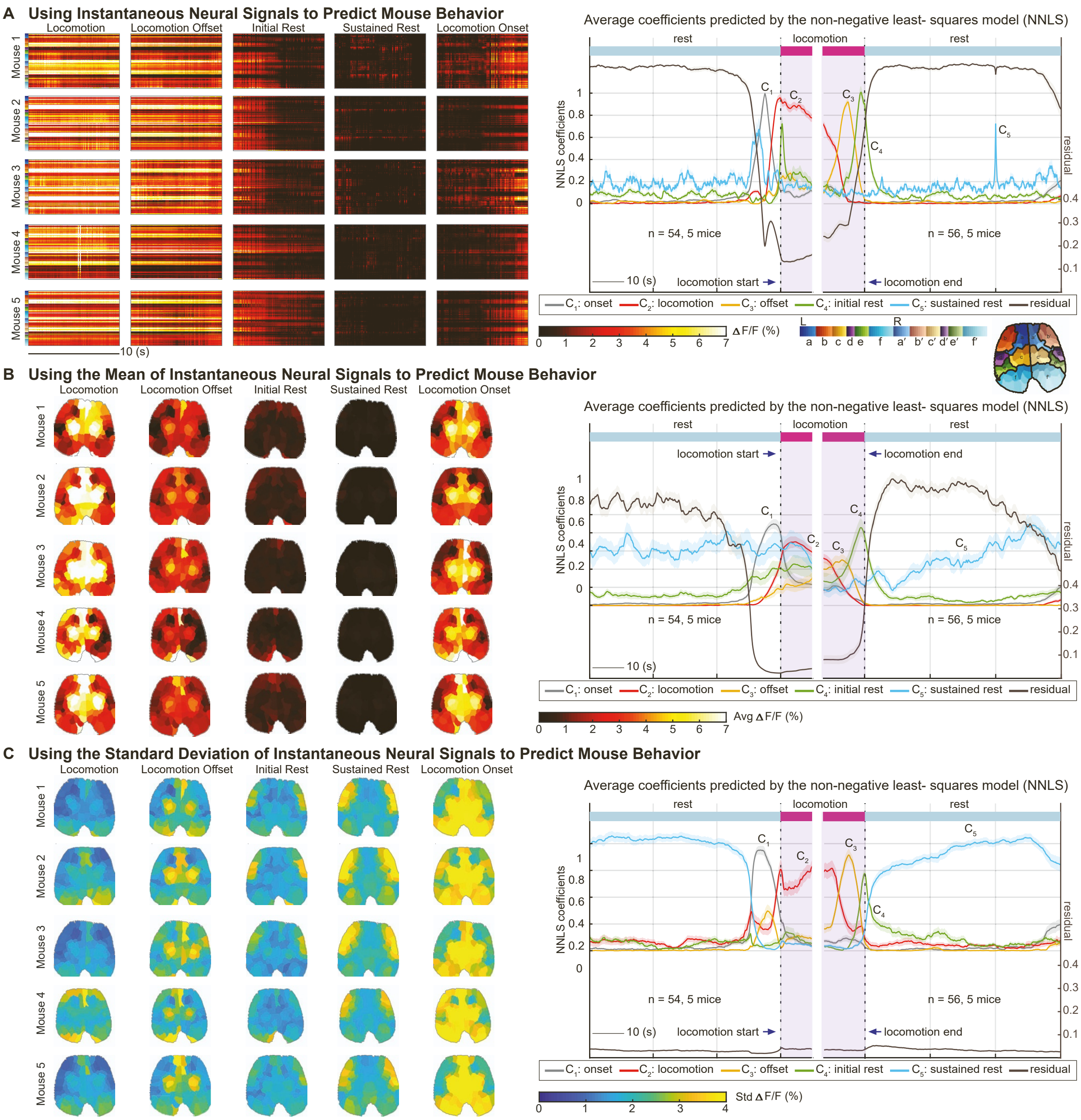

Supplemental Figure S5. Using neural signatures to predict mouse behavior. Related to **Figure 3.** Instead of correlation maps, **(A)** instantaneous neural signals, **(B)** the average of neural signals and **(C)** their standard deviation were used to represent the brain states. These neural signatures were extracted over 10-second windows time-locked to five distinct behaviors shown in **Figure 3A**: locomotion, locomotion offset, initial rest (immediately after locomotion end), sustained rest (40-second rest after locomotion end) and locomotion onset. The rectangular maps in (A) show instantaneous neural signals with 92 rows corresponding to ROIs. The cortex maps in (B) depict the region-resolved mean of neural signals, and the cortex maps in (C) illustrate the region-resolved standard deviation of neural signals. These neural signatures were separately used as a set of bases for non-negative least-squares (NNLS) fits. For each feature, the average coefficients predicted by NNLS over the temporal windows of interest (mean±SEM). The bouts were aligned around locomotion onset (n = 54) and locomotion offset (n = 56) across all five mice.

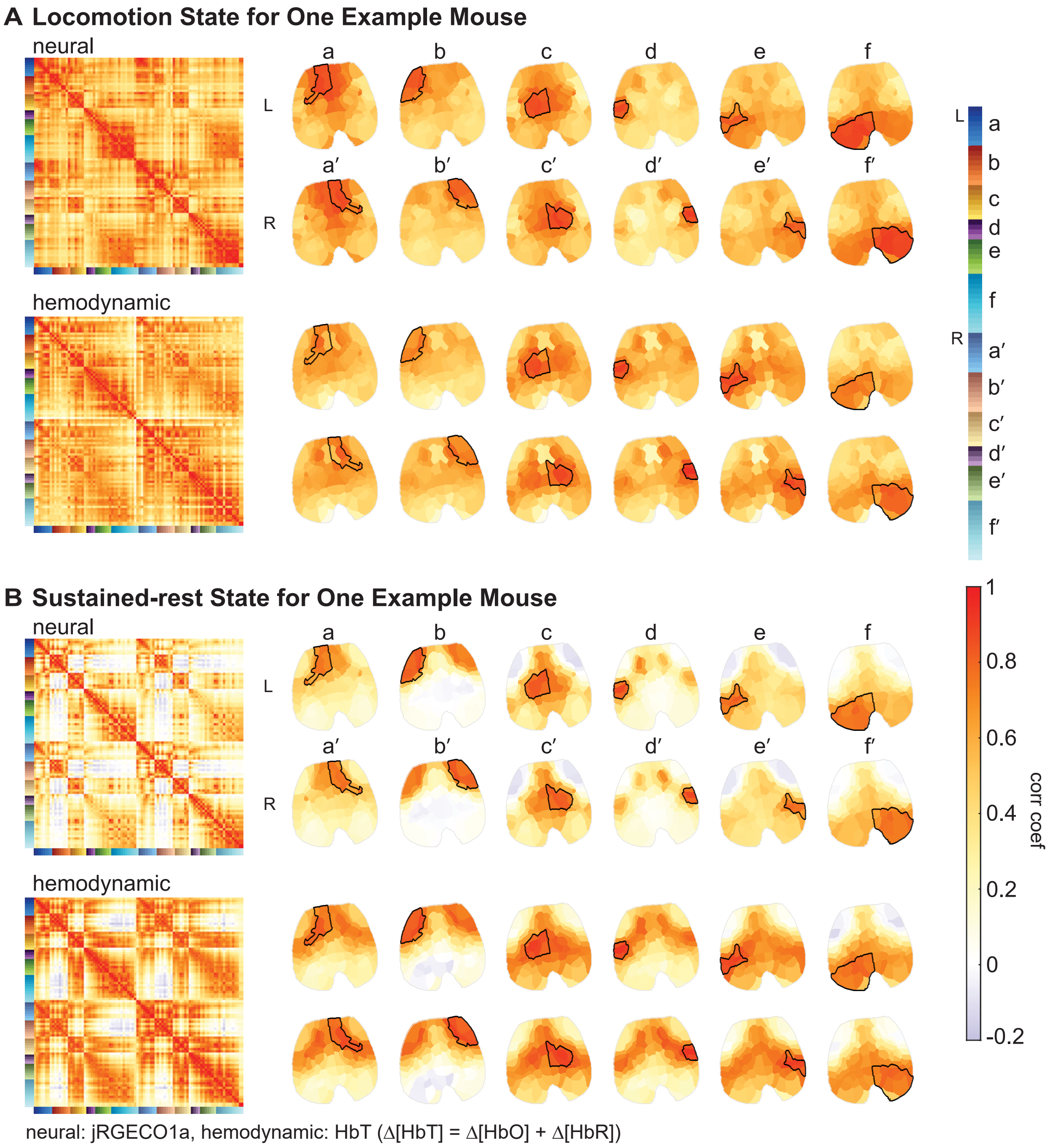

Supplemental Figure S6. Locomotion and sustained-rest states for both neural and hemodynamic activity. Related to **Figure 7.** **(A)** Locomotion state for one example mouse. The correlation maps were computed and averaged over 10-second windows in the middle of locomotion bouts (n = 36). **(B)** Sustained-rest state for the same example mouse. The correlation maps were computed and averaged over 10-second resting windows starting at 40 seconds after locomotion offset (n = 63).

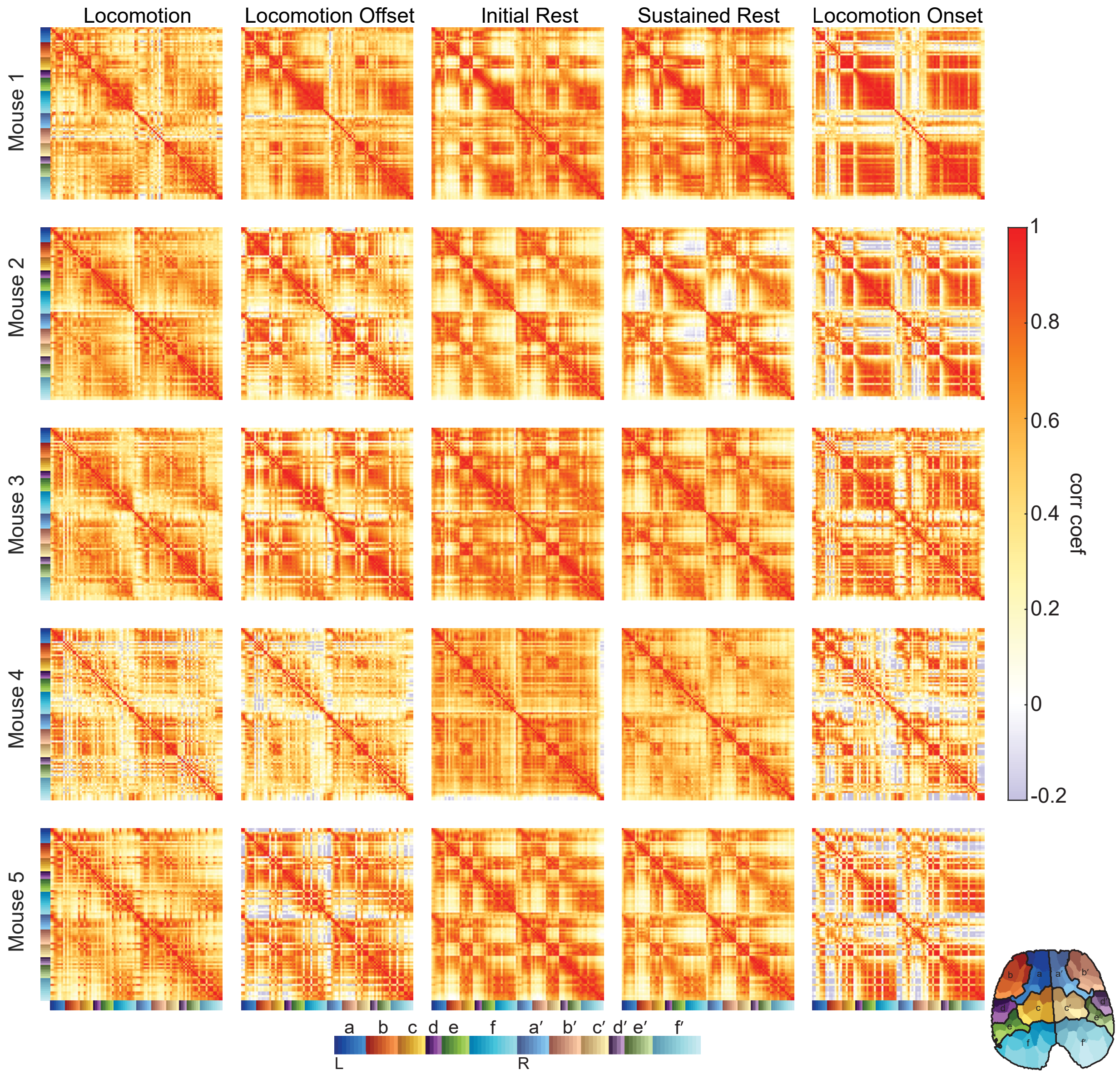

Supplemental Figure S7. Behavior-driven hemodynamic brain states for every mouse. Related to **Figure 7.** The total hemoglobin (Δ[HbT] = Δ[HbO] + Δ[HbR]) signals were time-locked to five distinct behavior windows 10 seconds long, shown in **Figure 3A**: locomotion, locomotion offset, initial rest (immediately after locomotion end), sustained rest (40-second rest after locomotion end) and locomotion onset. The correlation maps were computed as pairwise Pearson correlation coefficients between the HbT signals of 92 ROIs within each window. The brain states were defined as the average of the correlation maps across all epochs within each behavior window. Note that while these hemodynamic locomotion, initial and sustained rest states are quite consistent across mice with neural correlation maps shown in **Supplemental Figure S4**, the hemodynamic locomotion-onset and locomotion-offset states for Δ[HbT] are more variable. We believe that this variability is caused by the differing effects of temporal smoothing caused by neurovascular coupling, since both of these states span sharp transitions between rest and locomotion, or locomotion and rest. Nevertheless, on a mouse-by-mouse basis, these onset and offset correlation states were sufficient to provide behavioral-state predictions from hemodynamic data using NNLS as shown in **Figure 7.**

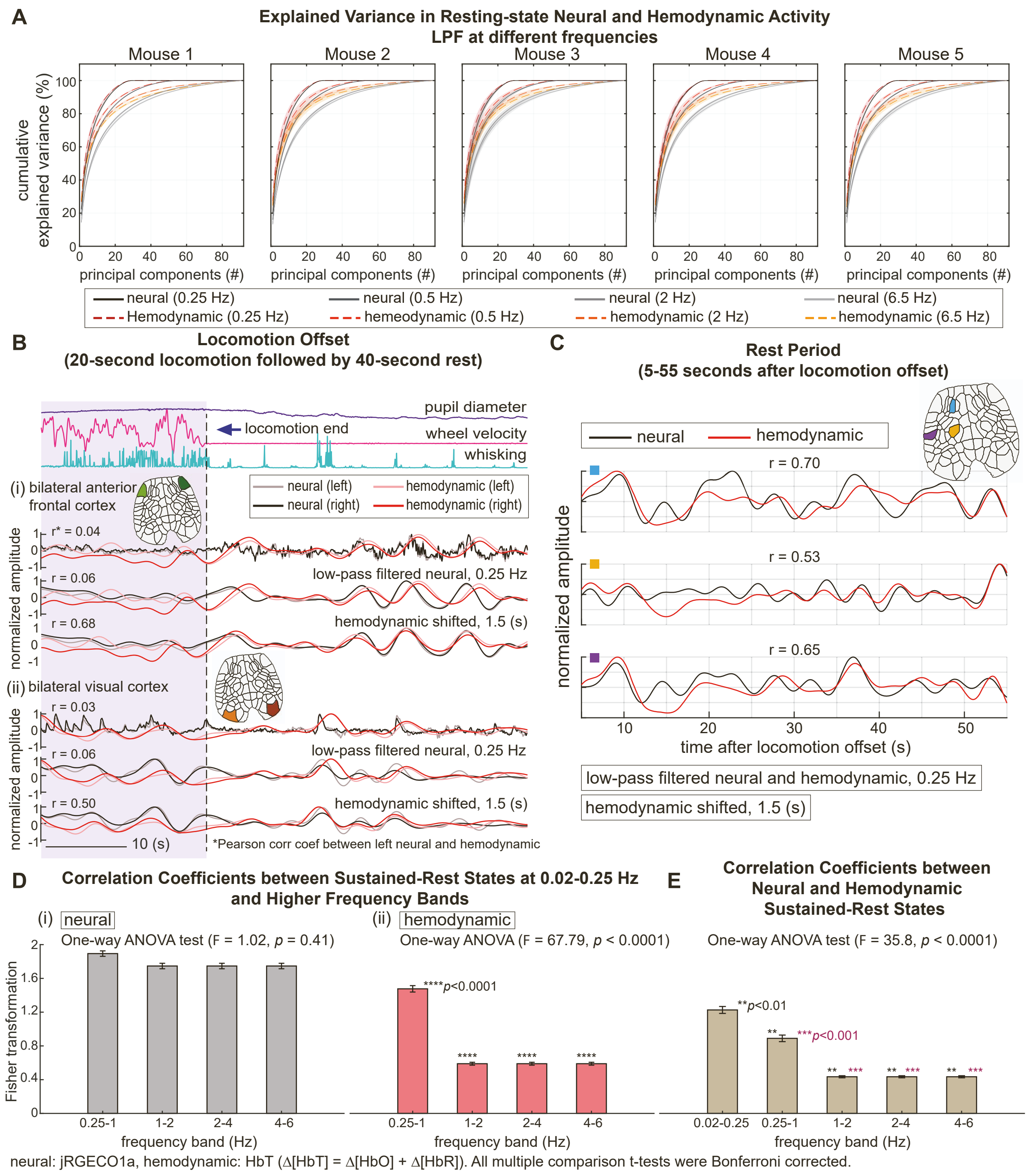

Supplemental Figure S8. Comparing neural and hemodynamic data. Related to **Figure 7. (A)** Explained variance in neural activity (solid lines) and hemodynamic activity (dashed lines) under different low-pass cut-off frequencies. The principal component analysis was performed on 50-second resting activity starting five seconds after locomotion offset and averaged over multiple epochs shown as mean±SD. The cumulative variance explained by principal components is shown in red for hemodynamic activity. The other colors indicate neural activity. **(B)** Neural and hemodynamic activity for one example of a 20-second locomotion bout followed by 40-second rest. The neural and hemodynamic time courses were extracted from two bilateral example ROIs within (i) the anterior lateral frontal cortex and (ii) the visual cortex. In both (i) and (ii), the neural and hemodynamic signals in the upper plot have frequency information up to 6.5 Hz and 0.25 Hz, respectively. The neural signals in the middle plot were low-pass filtered (0.25 Hz), and the hemodynamic signals in the lower plot were shifted (1.5 seconds). The r values show the Pearson correlation coefficients between the neural and hemodynamic signals of the left example ROIs. **(C)** Neural and hemodynamic time courses of three example ROIs for a rest period. Both neural and hemodynamic signals were low-pass filtered at 0.25 Hz, and hemodynamic signals were temporally shifted with a delay of 1.5 seconds. **(D)** The correlation between the resting state at 0.02-0.25 Hz and the resting states at higher frequency bands for (i) neural and (ii) hemodynamic activity (see **Figure 7G**). **(E)** The correlation between neural and hemodynamic resting states at different frequency bands. In (D) and (E), Fisher's transformations of Pearson correlation coefficients were averaged across five mice (mean±SEM) and compared using one-way ANOVA and multiple comparison t-tests ($p < 0.05$).

Supplemental References:

S1. Wang, Q., S.-L. Ding, Y. Li, J. Royall, D. Feng, P. Lesnar, N. Graddis, M. Naeemi, B. Facer, and A. Ho, *The Allen mouse brain common coordinate framework: a 3D reference atlas.* Cell, 2020. **181**(4): p. 936-953. e20.

Supplemental Movies:

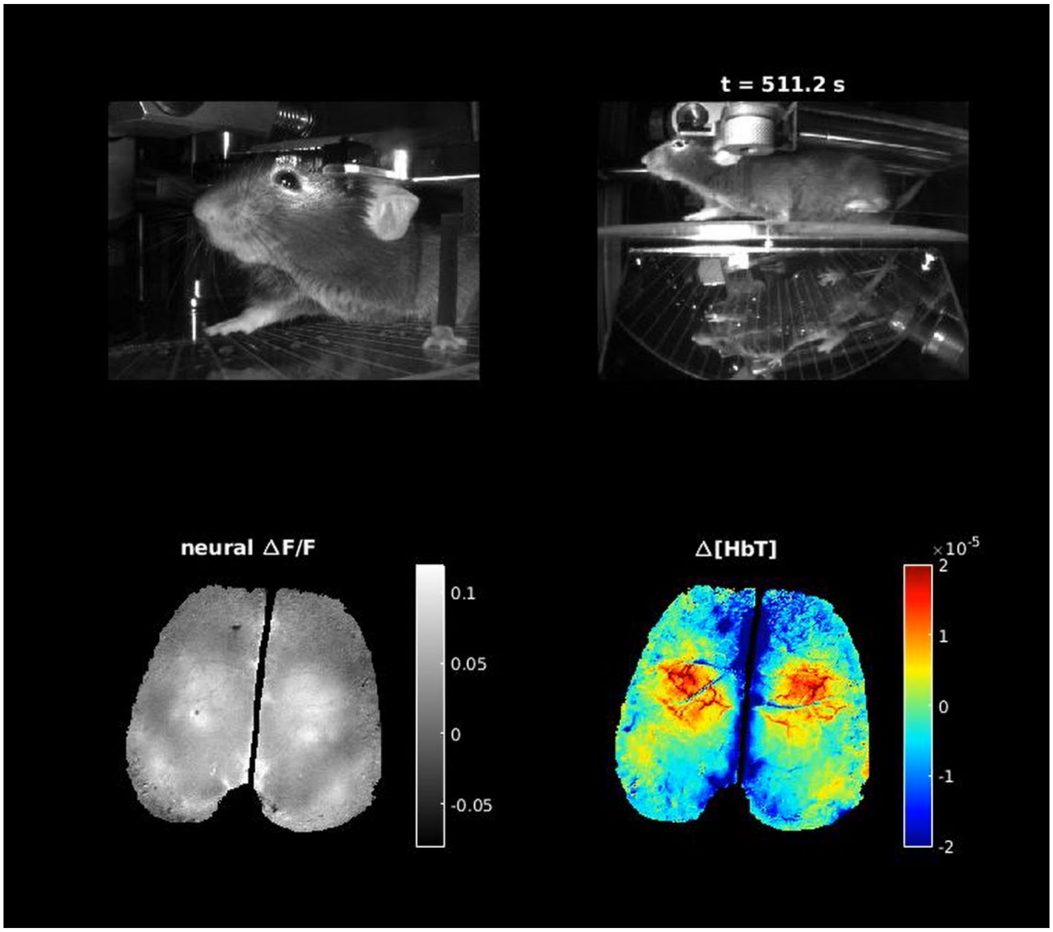

Supplemental Movie M1. Real-time data and behavior. Related to **Figure 1.** The top panels show behavior cameras for the same mouse and run as the epoch in Figure 1. The bottom left panel shows corrected jRGECO1a data corresponding to calcium activity in neurons across the dorsal cortex. The bottom right panel shows corresponding changes in total hemoglobin. The movie is played at ½ speed to enable appreciation of fast events.

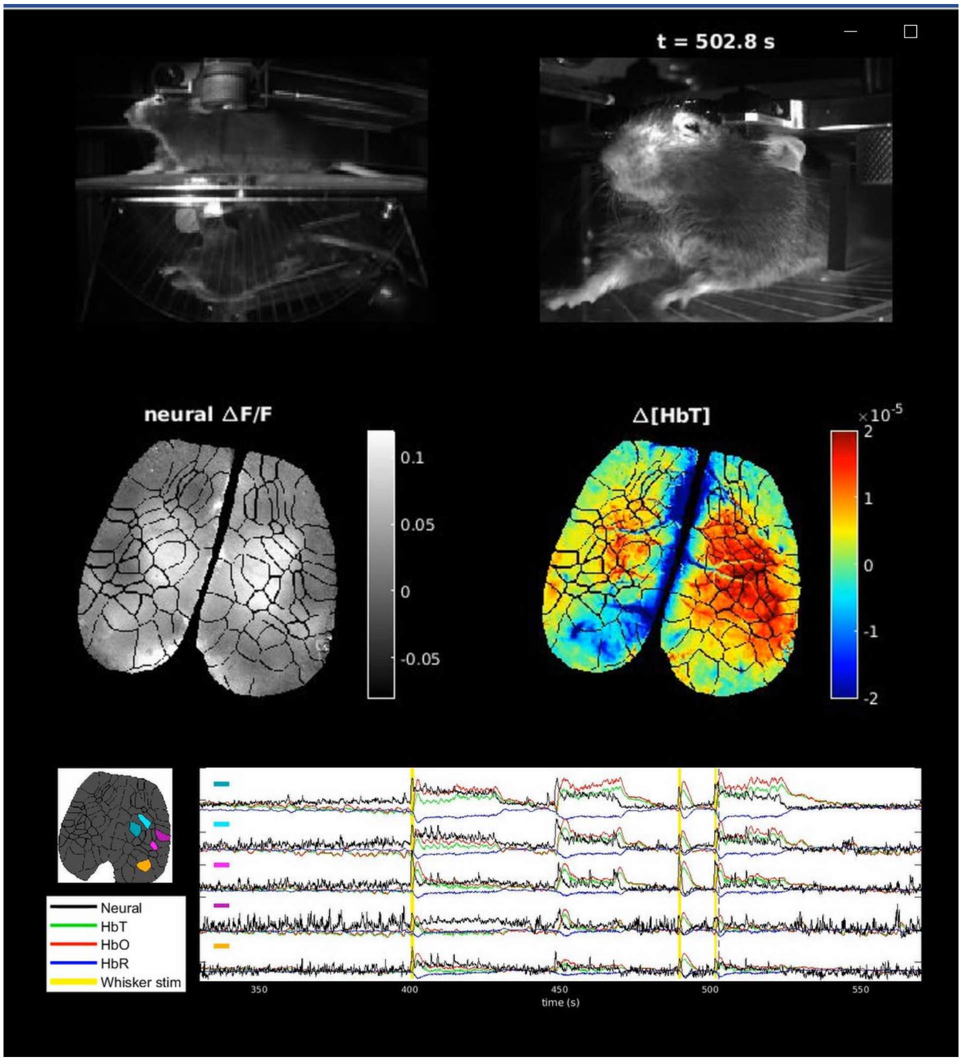

Supplemental Movie M2. Real-time data and behavior during random whisker stimulation. Related to **Figure 1** and **Figure 2.** The top panels show behavior cameras for the same mouse and run as the epoch in **Supplemental Figure S2**. The middle left panel shows corrected jRGECO1a data corresponding to calcium activity in neurons across the dorsal cortex. Outlines of the topographic map of regions of interest for this mouse are overlaid. The middle right panel shows corresponding changes in total hemoglobin. The bottom panel shows the full tie-course for neural, oxy-, deoxy- and total hemoglobin for the five regions indicated in the map to the left. Vertical yellow bars indicate whisker stimulation and the vertical dotted line indicates the current frame. The movie is played at 1x speed.

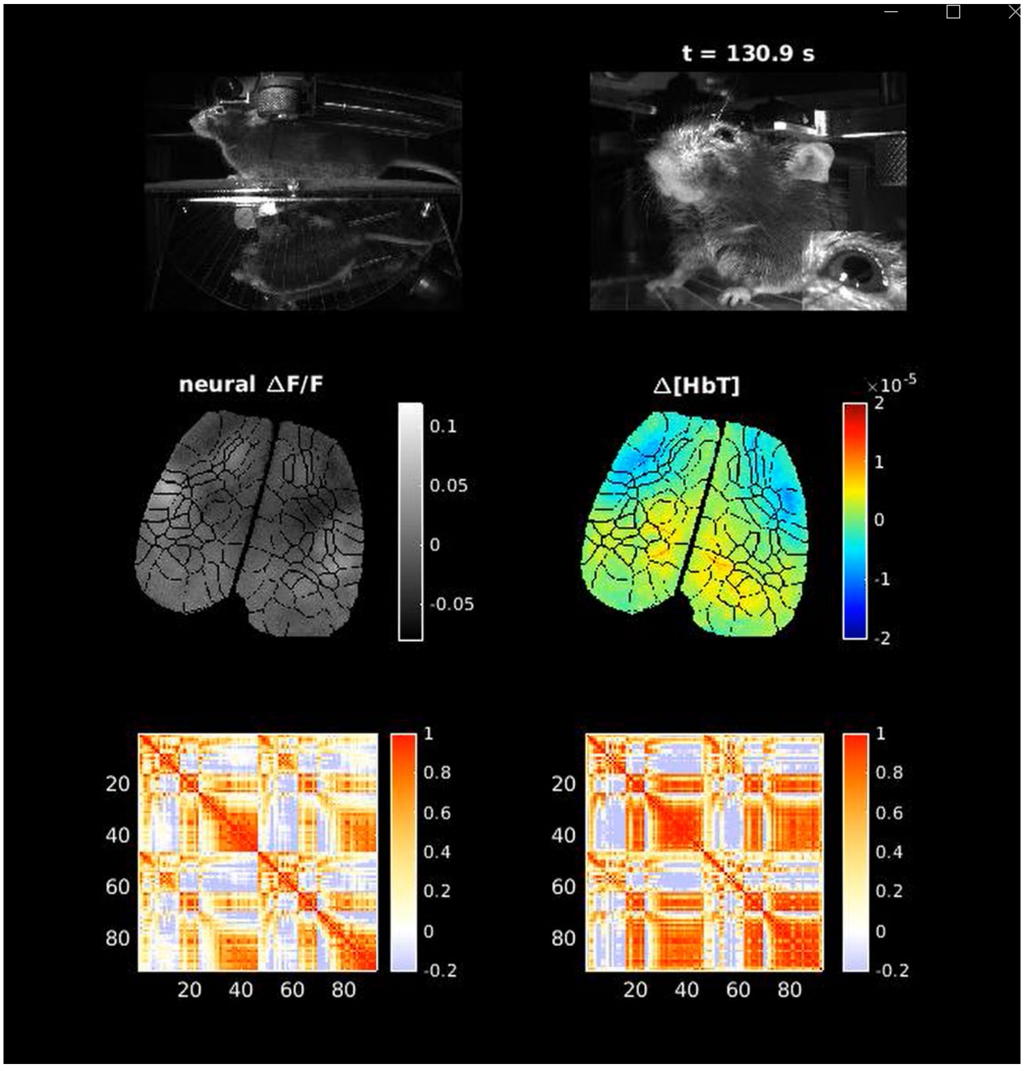

Supplemental Movie M3. Real-time data and behavior with dynamic correlation maps. Related to **Figure 2** and **Figure 7.** Example data for the same mouse as in **Supplemental Figure S2**, but for a session without whisker stimulation. The top panels show behavior cameras. The top right panel inset shows a zoomed-in view of the mouse's pupil. The middle left panel shows corrected jRGECO1a data corresponding to neuronal calcium activity across the dorsal cortex. Outlines of the topographic map of regions of interest for this mouse are overlaid. The middle right panel shows corresponding changes in total hemoglobin (HbT). The bottom panels show dynamically changing correlation maps for data over the subsequent 10-second window for neural (left) and HbT (right) data. The movie is played at 1x speed.

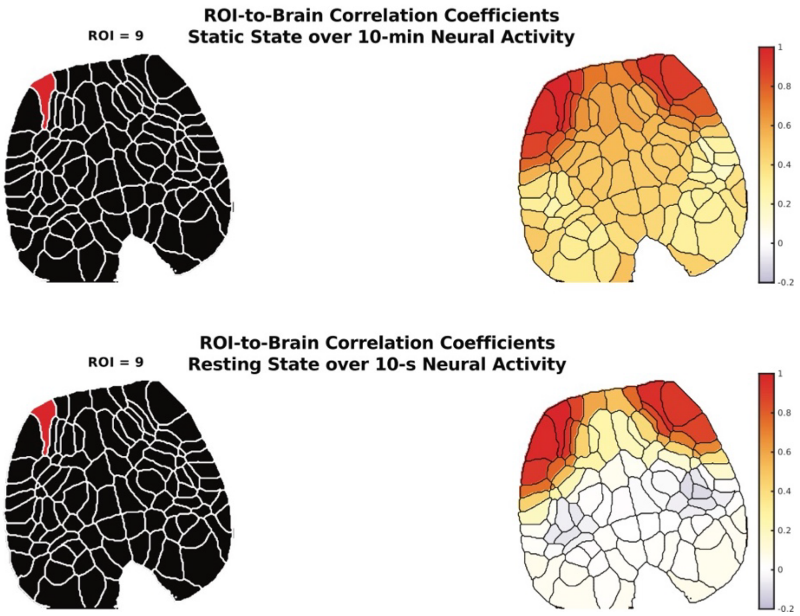

Supplemental Movie M4. Full representation of average 92x92 correlation maps. Top is related to **Figure 2C** and bottom is related to **Figure 3B.** The movie steps through correlation maps (right) for each individual ROI (left). The top row shows averaged maps over a full 10-minute epoch that includes rest and locomotion across all mice. The bottom row shows averaged maps for a 10-second window during sustained rest for one example mouse. This visualization is dimensionally reduced by summing sub-sets of regions in representations shown in the main figures.
